# Glyphosate: Hepatotoxicity, Nephrotoxicity, Hemotoxicity, Carcinogenicity, and Clinical Cases of Endocrine, Reproductive, Cardiovascular, and Pulmonary System Intoxication

**DOI:** 10.1021/acsptsci.4c00046

**Published:** 2024-04-08

**Authors:** Jarosław Mazuryk, Katarzyna Klepacka, Włodzimierz Kutner, Piyush Sindhu Sharma

**Affiliations:** † Department of Electrode Processes, Institute of Physical Chemistry, Polish Academy of Sciences, 01-224 Warsaw, Poland; ‡ Bio & Soft Matter, Institute of Condensed Matter and Nanosciences, Université catholique de Louvain, 1 Place Louis Pasteur, 1348 Louvain-la-Neuve, Belgium; § ENSEMBLE^3^ sp. z o. o., 01-919 Warsaw, Poland; ∥ Faculty of Mathematics and Natural Sciences. School of Sciences, Cardinal Stefan Wyszynski University in Warsaw, 01-938 Warsaw, Poland; ⊥ Functional Polymers Research Team, Institute of Physical Chemistry, Polish Academy of Sciences, 01-224 Warsaw, Poland

**Keywords:** carcinogenicity, cardiopulmonary system, endocrine and reproductive systems, glyphosate-based herbicide, hepatotoxicity and nephrotoxicity, multiorgan toxicity

## Abstract

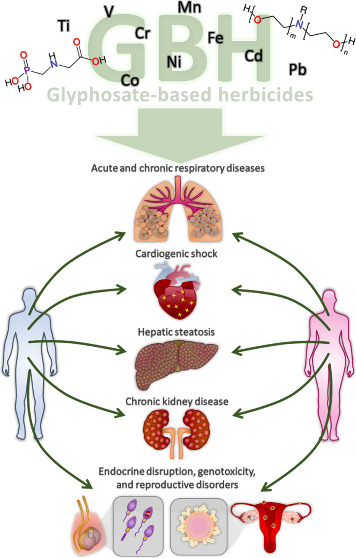

Glyphosate (GLP) is an active agent of GLP-based herbicides (GBHs), i.e., broad-spectrum and postemergent weedkillers, commercialized by Monsanto as, e.g., Roundup and RangerPro formulants. The GBH crop spraying, dedicated to genetically engineered GLP-resistant crops, has revolutionized modern agriculture by increasing the production yield. However, abusively administered GBHs’ ingredients, e.g., GLP, polyoxyethyleneamine, and heavy metals, have polluted environmental and industrial areas far beyond farmlands, causing global contamination and life-threatening risk, which has led to the recent local bans of GBH use. Moreover, preclinical and clinical reports have demonstrated harmful impacts of GLP and other GBH ingredients on the gut microbiome, gastrointestinal tract, liver, kidney, and endocrine, as well as reproductive, and cardiopulmonary systems, whereas carcinogenicity of these herbicides remains controversial. Occupational exposure to GBH dysregulates the hypothalamic–pituitary–adrenal axis, responsible for steroidogenesis and endocrinal secretion, thus affecting hormonal homeostasis, functions of reproductive organs, and fertility. On the other hand, acute intoxication with GBH, characterized by dehydration, oliguria, paralytic ileus, as well as hypovolemic and cardiogenic shock, pulmonary edema, hyperkalemia, and metabolic acidosis, may occur fatally. As no antidote has been developed for GBH poisoning so far, the detoxification is mainly symptomatic and supportive and requires intensive care based on gastric lavage, extracorporeal blood filtering, and intravenous lipid emulsion infusion. The current review comprehensively discusses the molecular and physiological basics of the GLP- and/or GBH-induced diseases of the endocrine and reproductive systems, and cardiopulmonary-, nephro-, and hepatotoxicities, presented in recent preclinical studies and case reports on the accidental or intentional ingestions with the most popular GBHs. Finally, they briefly describe modern and future healthcare methods and tools for GLP detection, determination, and detoxification. Future electronically powered, decision-making, and user-friendly devices targeting major GLP/GBH’s modes of actions, i.e., dysbiosis and the inhibition of AChE, shall enable self-handled or point-of-care professional-assisted evaluation of the harm followed with rapid capturing GBH xenobiotics in the body and precise determining the GBH pathology-associated biomarkers levels.

## 1 Introduction

Glyphosate (GLP), *N*-(phosphonomethyl)glycine, is an active ingredient (ActI) of the most common herbicides used in contemporary agriculture, forestry, industrial weed control, lawn, garden, and aquatic environments. By slow, reversible, and competitive inhibition of 5-enolpyruvynyl-shikimate-3-phosphate synthase (EPSPS, EC 2.5.1.19), responsible for the biosynthesis of aromatic amino acids in plants^1^ and several strains of bacteria, yeast, algae, and fungi, GLP acts as one of the most effective and broad-spectrum agrochemicals ever produced.^2,3^ First synthesized in 1950, then commercialized in the herbicide market in 1974, GLP has become the most widely used, postemergent, nonselective weedkiller worldwide.^4^

Agricultural use of genetically engineered (GE) GLP-tolerant crops, commercial GLP-surfactant (GLP-SH), and GLP-based herbicides (GBHs), often used as Roundup or RangerPro commercial formulations, applied within the so-called “green burn-downs” has modernized the harvest, weed, and herbicide management. In recent years, GBH usage has increasingly been withdrawn in EU, the U.S.A., and then worldwide.^5^ Significant causes include environmental pollution of GBH, the development of herbicide-resistant weeds (superweeds) and microorganisms (superbugs), as well as overconsumption of GE organisms and GBH-contaminated products.^6−8^ Concerningly, the relevant epidemiological and environmental contamination risk and human toxicity are driven by the growing reports on the health issues among farmers and occupational workers of GBH factories.^9,10^

The most poisonous xenobiotics present in GBHs are GLP metabolites, e.g., (aminomethyl)phosphonic acid (AMPA), dyes, antifoaming agents, inert ingredients, and adjuvants, e.g., ethoxylates and polyoxyethyleneamine (POEA), more toxic than GLP itself,^11−14^ and ppb traces of heavy metals, including chromium, cobalt, lead, or nickel.^15^ Notably, the toxicity of these coformulants is diverse and variant. According to the insightful review of pesticide variability,^16^ ∼750 different GBH formulations on the market contain various combinations of the GLP active principle and coformulants. Moreover, because of the legal regulations, the total composition of GBH is classified as confidential commercial information.^17^ Hence, in commercial brochures and scientific reports, this heterogeneity of ingredients is usually simplified and defective, which may cause confusion and potential risk, even though the toxicological details of each ingredient included are well-described in the medical literature.

The current review article presents the poisoning with GLP, GLP-SH, and GBHs impact on the endocrine, reproductive, and cardiopulmonary systems, also mentioning its hepato- and nephrotoxic consequences. Besides, we introduce the potential carcinogenic effects of this poisoning. Ailments of the gastrointestinal tract and nervous system, including gut dysbiosis and the dysregulation of microbiota-gut-brain-axis, are discussed in our review article concerning GLP and GBH toxicity,^18^ whereas an extensive summary of novel technologies applied to GLP sensing is presented in our recent critical review.^19^

## 2 Environmental and Human Toxicity of Glyphosate

GLP and GBHs are remarkably poisonous to the gut microbiome (gut microbiota, GM)^20,21^ and the neurological system^22−25^ by affecting the microbiota-gut-brain (MGB) axis and GLP-mediated inhibition of acetylcholinesterase (AChE) and cholinergic neurotransmission.^22,26−29^ GBHs have also been cautioned as endocrine system disruptors (ESDs).^30−34^ However, over decades, this topic has become exceptionally controversial^35,36^ (Tables 1 and 2). Regardless of several studies demonstrating the dysregulating activity of GLP/GBH on the hypothalamic-pituitary-adrenal (HPA) axis, hypothalamic-pituitary-peripheric glands (HPP) axes,^23,37^ steroidogenesis,^38−42^ or reproductive system,^37,43−49^ this ESD activity has been questioned by the EPA’s Endocrine Disruptor Screening Program (EDSP) and, subsequently, by the European Food Safety Authority (EFSA). In 2015, the EPA and EFSA eventually declined the direct interaction of GLP with estrogen, androgen, and thyroid (EAT) pathways involving modes of action.^50−52^ Because of the absence of the EPSP-metabolic pathway in vertebrates and the quick elimination of GLP, after its absorption and accumulation, from mammals, the GLP half-life time (∼5 to 10 h) is relatively short. Therefore, GLP is expected to be slightly or minimally toxic. However, many distressing reports have recently pointed to the GLP and GBH pathogenesis, indicating both direct and indirect influences on the intestinal tract and GM^25,53,54^ and reproductive system,^43,55^ as well as neurotoxicity and MGB-associated neurological disorders,^20,21^ and carcinogenicity^43^ (Table 3).

**Table 1:** Preclinical Studies on GLP and GBH Toxicity on Liver, Kidneys, and Endocrine and Reproductive Systems^a^

GBH	GLP	ref.
*Endocrine system disruption and reprotoxicity*		
Roundup Bioflow (0.36 g/mL of GLP), in boars, decreased sperm motility (≥5 μg/mL; GLP-equivalent concentration), mitochondrial activity (≥25 μg/mL), and sperm viability and acrosome integrity (≥100 μg/mL).	360 μg/mL GLP impaired sperm motility, viability, mitochondrial activity, and acrosome	(11)
Roundup Original (homologation No. 00898793, 360 g/L GLP), at 0.036 g/L in prepubertal rat testis, caused Ca^2+^ overload, oxidative stress, and necrosis of Sertoli cells.	0.036 g/L, only Ca^2+^ overload.	(12)
Roundup (41% GLP-IPA salt, effective GLP conc. 30%), applied at 10–40 mg/kg in the basal diet of weaned piglets, increased SOD and GPx levels in the uterus).	n. m.	(23)
RoundUp Flex (480 g/L GLP, 43.8% *w*/*w*), applied to Japanese quails at 12–20 mg GLP/kg bw/day, impaired embryonic development and caused lipid-related brain damage.	n. m.	(24)
Roundup Maxload (48%, *w*/*v* GLP salt); 1% Roundup equals 50 mg/kg/day of GLP; applied at 0.1, 0.25, 0.50, 0.75, 1.0% to pregnant mice; in the juvenile offspring Roundup caused ASD-like behavioral abnormalities, higher levels of sEH in the PFC, hippocampus, and striatum, and decreased levels of epoxy-fatty acids in the blood, PFC, hippocampus, and striatum, and anomalies in composition of gut microbiota and short-chain fatty acids in fecal samples.	n. m.	(25)
Roundup, applied to zebrafish at 0.065 and 0.5 mg/L, impaired the exploratory behavior more than 0.065 and 0.5 mg/L GLP, whereas at 0.065 and 0.5 mg/L, Roundup caused a higher memory impairment than 0.065 and 0.5 mh/L GLP.		(28)
Roundup Bioflow, at 1.75 mg/kg bw/day, in male and female rats, induced higher endocrine effects and altered reproductive developmental parameters than 1.75 mg/kg bw/day GLP.	n. m.	(30)
Roundup Original DI [445 g/L GLP, which corresponds to 370 g/L (37% m/v)], applied at 0.5% GLP-Roundup to mice, decreased spermatogenesis and disruptions in hypothalamus-pituitary-testicular axis regulation in the F1 offspring.	n. m.	(33)
Roundup 3 Plus (229 g/L GLP-IPA, 170 g/L GLP equivalent) and GLP effects in mice:		(34)
• the spermatozoa number decreased by 89% and 84% in 0.5 and 5 mg/kg/day of Roundup and GLP groups, respectively.		
• the undifferentiated spermatogonia numbers decreased by 60% in 5 mg/kg/day GLP group, possibly due to the altered expression of genes involved in germ cell differentiation (Sall4 and Nano3) and apoptosis (Bax and Bcl2).		
• in 8 m.o. animals, a decreased testosterone level was observed in Roundup groups.		
• perinatal exposure to GLP is reprotoxic to young animals, contrary to Roundup exposure which is less toxic in the short term but shows its effect in the long term in testosterone levels and testis weight.		
Roundup (GLP-IPA salt) and GLP, applied as 0.5% GLP or 0.5%-GLP Roundup to pregnant mice; GBH and GLP effects:		(37)
• both formulations decreased the body weight gain and ovary and liver weight;		
• increases in atretic follicles, interstitial fibrosis and decreased mature follicles;		
• alterations in the serum concentrations of both progesterone and estrogen;		
• changes in the expression of GnRH, LHR, FSHR, 3β-HSD and Cyp19a1 genes at the HPO axis;		
• increases in the activity of T-AOC, CAT and GSH-Px, as well as the MDA content in both the serum and ovary;		
• the sex ratio was significantly altered by GLP.		
Roundup Grand Travaux Plus (no. 2020448; 450 g/L GLP), applied at 0.50% to rats, increased aromatase mRNA and protein levels, Gper1 expression and a slight modification of BTB markers; increased abnormal sperm morphology and decreased the expression of protamine 1 and histone 1 testicular in epididymal sperm	n. m.	(40)
n. m.	GLP (25 mg/kg bw/day) applied to rats; negligible impacts on F0 and F1 generation rats; increases in pathologies in the F2 and F3 generation and transgenerational offspring, including prostate disease, obesity, kidney disease, ovarian disease, and birth abnormalities, and altered differential DNA methylation regions (DMRs).	(247)
Roundup Full II (54 g of GLP per 100 mL of GBH), applied at 2 mg/kg/day to rats, increased luminal epithelial height and stromal nuclei density, caused the luminal and glandular epithelium hyperplasia in 43% of GBH-exposed animals, increased the E2-induced cell proliferation, decreased membranous and cytoplasmic expression of CTNNB1 in luminal and glandular epithelial cells and increased WNT7A expression in the luminal epithelium.	n. m.	(46)
Commercial GBH (66.2% of GLP salt, equivalent GLP is 54%), applied at 2 mg/kg/day to rats, caused a significant increase in the number of resorption sites, altered decidualization response, decreased expression of estrogen and progesterone receptors, and dysregulated Nr2f2, Bmp2, HOXA10, and Ki67 levels.	n. m.	(47)
Roundup Full II (54 g of GLP per 100 mL of GBH), applied at 2 mg/kg/day to rats, increased Wnt5a and catenin expression in luminal epithelium (LE), and increased Wnt5a and catenin expression in subepithelial stroma but decreased catenin expression in glandular epithelium.	n. m.	(48)
Magnum Super (66.2% of GLP salt, equivalent to 54% w/v of GLP) and GLP, applied to rats at 2 mg of GLP/kg/day, caused induced preimplantation losses in F1 offspring, higher 17β-estradiol serum levels, increased uterine ERα protein expression, and downregulated Hoxa10 and Lif genes, whereas only GLP decreased PR mRNA expression.		(49)
Magnum Super II (equivalent to 54% w/v GLP), applied at 350 mg GLP/kg bw/day, upregulated the expression of total ERα mRNA, decreased DNA methylation, enriched histone H4 acetylation and histone H3 lysine 9 trimethylation (H3K9me3) and decreased H3K27me3.	n. m.	(69)
Roundup Full II (54 g of GLP per 100 mL of GBH), applied at 2 mg GBH/kg bw to male rats, caused greater development of the mammary gland with increased stromal collagen organization and terminal end buds, mast cell infiltration, proliferation and ESR1 expression.	n. m.	(70)
Magnum Super II (54 g of GLP per 100 mL of GBH), applied at 3.5 or 350 mg GBH/kg bw/day to rats; GBH3.5 caused higher AR protein expression, whereas GBH350 decreased less developed mammary gland, lower proliferation index and slightly increased PRL serum levels. GHB3.5 decreased only ESR1-OS expression, whereas GBH350 affected ESR1-O, OT and E1 expression; both GBHs reduced ESR1 expression and altered the abundance of ESR1 transcripts.	n. m.	(73)
Roundup (48 g GLP-IPA per 100 mL; equivalent to 35.6% w/v GLP), applied at 0.38% GLP (1% Roundup), equivalent to 50 mg of GLP/kg/day, to pregnant mice, upregulated 55 and downregulated 19 miRNAs in the PFC of mouse offspring, causing changes neurogenesis, neuron differentiation, and brain development.	n. m.	(74)
Roundup (equivalent to 35.6% w/v of GLP), applied at 50 mg of GLP/kg/day to mice, altered 663 circRNAs associated with stress-associated steroid metabolism pathways in the hippocampus.	n. m.	(75)
Commercial GBH, sprayed at 0.25 ppm, 0.87–1.13 ppm to farmlands, was found in lungs (0.4–80 μg/mL), hearts (0.15–80 μg/mL), and in muscles (4.4–6.4 μg/mL) of livestock animals, causing ear atrophy, spinal and cranial deformations, cranium hole in head and leg atrophy; one-eye syndrome, trunk disappearance, elephant tongue, testes presence in females, and swollen belly and fore gut and hind gut.	n. m.	(76)
Roundup, applied at 10, 50, 100, and 250 mg/kg bw/day to rats; at 10 mg/kg bw/day dose, GBH reduced circulatory corticosterone, cholesterol receptor (low-density lipoprotein receptor), de novo cholesterol synthesis enzyme, hormone-sensitive lipase, steroidogenic acute regulatory protein (StAR) mRNA and phosphorylated form, and circulatory ACTH and adrenal cortex levels of protein kinase A (PKA) activity were reduced; apoptosis was evident at 250 mg/kg bw/day, but not at 10 mg/kg bw/day dose.	n. m.	(79)
Roundup (360 g/L of GLP), applied at 50, 150, or 450 mg/kg GLP-Roundup to rats, induced adverse reproductive effects on male offspring rats, decreased sperm number per epididymis tail and in daily sperm production during adulthood, increased the percentage of abnormal sperms, decreased the serum testosterone level at puberty, and signs of individual spermatid degeneration during both periods.	n. m.	(83)
Roundup Transorb (480 g/L GLP), applied at 5, 50, or 250 mg/kg to rats, changed in the progression of puberty in a dose-dependent manner, reduced the testosterone production, changed seminiferous tubules’ morphology, decreased the epithelium height, and increased the luminal diameter, and decreased the concentrations of testosterone serum.	n. m.	(84)
n. m.	GLP, applied at 50 and 500 mg/kg, decreased the average daily feed intake at the dose of 50 mg/kg, and the weight of the seminal vesicle gland, coagulating gland and the total sperm count at the dose of 500 mg/kg.	(85)
Roundup Transorb, applied at 5 mg/kg/day or 50 mg/kg/day to rats, decreased concentrations of TSH, dysregulated hypothalamic and pituitary gene expression, thus impacting the thyrotrophic axis.	n. m.	(82)
Roundup (equal molar for GLP) and GLP, applied at 1.75 mg/kg bw/day to rat dams and their pups, increased homocysteine levels		(53)
Kalach 360 SL, dosed at 126 and 315 mg/kg to rats, perturbed bone metabolism (calcium and phosphorus), disturbed morphological structure and thyroid cells function, decreased triiodothyronine and thyroxine.	n. m.	(92)
Roundup Transorb (480 g/L GLP), applied at 50 mg/kg to rats, increased sexual partner preference scores, testosterone and estradiol serum concentrations, changed the mRNA expression and protein content in the pituitary gland and the serum concentration of LH, and altered the height of the germinal epithelium of seminiferous tubules.	n. m.	(99)
Roundup Ultramax (67.9% w/w of GLP) and GLP, applied at 1 mg/L (active agent) to crabs, decreased the weight gain and muscle protein levels; in spermatophores from the vas deferens, Roundup decreased the sperm count, while abnormal spermatophores were observed either with GLP or with Roundup.		(100)
Roundup Original, applied at 50 and 100 mg/kg to male rats, decreased testosterone levels and the Sertoli cell number, increased the percentage of degenerated Sertoli and Leydig cells, decreased spermatids number and increased the epididymal tail mass, and decreased the diameter of seminiferous tubules. 100 mg/kg GBH decreased the round spermatids and increased the abnormal sperm morphology.	n. m.	(102)
Glyfonova 450 Plus (450 g/L GLP acid equivalent) and GLP, applied at 2.5 and 25 mg/kg bw/day to rats; GHB upregulated steroidogenic genes Cyp11a1 and Cyp17a1, whereas GLP caused no significant effects on testes or testosterone synthesis.		(104)
Commercial GBH (containing 662 mg/mL of GLP salt), applied at 0.5, 5, or 50 mg GBH/kg/day to rats; GBH0.5 increased the luminal epithelial cell height; all GBH doses downregulated ERα mRNA, whereas GBH0.5 diminished PR and C3 mRNA; GBH5 and GBH50 downregulated ERα protein expression in luminal epithelial cells, while the receptor was upregulated in the stroma. GBH upregulated ERβ (GBH0.5–50) and PR (GBH5) expressions in glandular epithelial cells.	n. m.	(107)
Roundup Full II (66.2% of GLP), applied at 2 mg/kg to rats, caused luminal epithelial hyperplasia, increased the stromal and myometrial thickness, altered PR, ER, Hoxa10, and the expression of proteins involved in uterine organogenetic differentiation.	n. m.	(108)
Roundup Full II, applied at 2 mg/kg/day to lambs, altered follicular dynamics, increased proliferation of granulosa and theca cells, decreased mRNA expression of FSHR and GDF9, and decreased cell proliferation in the uterus.	n. m.	(109)
Roundup Full II (66.2% GLP), applied at 2 mg GLP/kg/day to rats, caused a higher percentage of hyperplastic ducts and a fibroblastic-like stroma in the mammary gland, and a high expression of steroid hormone receptors in hyperplastic ducts.	n. m.	(110)
Roundup (no. 101667948A2 < 140720>) and GLP, applied 0.11 and 10 ng/mL; in granulosa cells, GLP stimulated the secretion of oestradiol while both herbicides increased and decreased oxytocin (OT) and progesterone secretion from luteal cells, respectively; only Roundup stimulated mRNA expression of the precursor of OT, and both herbicides decreased the secretion of prostaglandins from endometrial cells while they exerted no effect on the basal and OT-stimulated force of myometrial contractions.		(111)
n. m.	GLP applied at 1, 5, 10, and 100 mg/L to zebrafish, inhibited CA activity, caused the production of ROS, especially branchial regions, triggered cellular apoptosis and caused pericardial edema	(113)
Roundup Full II (66.2% GLP, GLY-RU), Panzer Gold (60.8% GLP, GLY-PZ), applied at 200, 400, and 800 mg/egg each to lizard embryos, decreased heterophils and lymphocytes populations, and antibody titres.	n. m.	(114)
Roundup, Kilo Max, and Enviro Glyphosate, applied to frogs at 0.3–1.3, 130–280, and 320–560 mg/mL of GLP equivalent, respectively, caused generalized teratogenic edema, cardiac and abdominal edema, improper gut formation and axial malformations. Roundup was the most toxic with a 96-h LC_50_ of 1.05 mg a.e/L compared with 207 mg a.e./L and 466 mg a.e./L for Kilo Max and Enviro Glyphosate, respectively.	n. m.	(115)
Herbicygon at 1% caused excessive lipid peroxidation and an overload of maternal and fetal antioxidant defense systems.	n. m.	(118)
Magnum Super II (66.2% of GLP salt, equivalent to 54% w/v GLP), applied at 2 or 200 mg GLP/kg bw/day to rats, caused delayed growth lower fetal weight and length and conjoined fetuses and abnormal limbs of F2 offspring, and higher placental weight and placental index.	n. m.	(119)
Roundup (BS 1096/98, 360 g/L GLP), applied at 500, 750, or 1000 mg GLP/kg to rats; effects: 50% mortality rate for dams treated with 1000 mg GLP/kg; skeletal alterations were observed in 15.4, 33.1, 42.0 and 57.3% of fetuses from the control, 500, 750, and 1000 mg GLP/kg groups, respectively.	n. m.	(120)
n. m.	GLP, applied at 24 or 35 mg/kg to tars, changed reflexes development, motor activity, and cognitive function in a dose-dependent manner and inhibited the Wnt5a-CaMKII signaling pathway in embryos.	(124)
n. m.	GLP, applied at 600, 400, 200, 100, 10, 5, 1, 0.5, 0.1, 0.01 mg/L to zebrafish; at concentrations higher than 10 mg/L, an obvious delay occurred in the epiboly process and body length, and eye and head area decreased; dose-dependent motoneuronal damage, chorion dysfunctionality,	(248)

a n. m.: not measured.

**Table 2:** Preclinical Studies on GLP and GBH Toxicity on Liver, Kidneys, and Cardiopulmonary Systems^a^

GBH	GLP	ref.
*Cardiotoxicity*		
n. m.	GLP, applied at 50 μg/mL to zebrafish, caused structural abnormalities in the atrium and ventricle, irregular heart looping, situs inversus, and decreased heartbeats by 48 h	(151)
Roundup (0.1, 1, 10, and 100 μM GLP) and GLP (0.1, 1, 10, and 100 μM), in human cardiomyocytes, effects: a significant effect on heart rate and depressive effect on ventricular contractility for 100 μM GBH by a dose-dependent blocking effect on cardiac calcium channel CaV1.2 with an IC50 value of 3.76 μM		(152)
GLP caused no significant cardiotoxicity.		
Roundup Original DI (no. 00513, GLP salt 445 g/L (370 g/L GLP equivalent), applied at 3.71, 6.19, and 9.28 mg GLP/ha to rats, caused fatty streaks.	n. m.	(154)
Roundup Ultra (ISO 9002, 36% GLP), applied at 2.5, 25, 50, 500, 5.000, and 20.000 ppm, to rats and rabbits; at 20.000 ppm, high incidence of conduction block was observed; different doses caused arrhythmias; at 50 ppm, APD90 cardiac inexcitability was shown.		(155)
Pure GLP, at 18 and 180 ppm, had no significant electrophysiological effects.		
*Pulmotoxicity*		
n. m.	GLP, at 33.33, 16.67, 8.33, 4.17, 2.08, 1.04, 0.52, and 0 mM; dose-dependent effects on melanin inhibition and oxidative stress; reduction of survival of caterpillars following infection with the fungus and decreased the size of melanized nodules formed in hemolymph, and the increase in the burden of the malaria-causing parasite in mosquitoes, altered uninfected mosquito survival, and perturbed the microbial composition of adult mosquito midguts.	(185)
n. m.	GLP, present in air samples at 22.59 ng/m^3^ dose, as well as pure GLP, administrated to mice by inhalation, increased levels of eosinophil and neutrophil counts, mast cell degranulation, and production of IL-33, TSLP, IL-13, and IL-5.	(187)
n. m.	GLP (1 μg/40 μL) and (1 μg/40 μL GLP + 0.5 μg/40 μL LPS), applied to male mice; the GLP and LPS mixture increased neutrophil counts, myeloperoxidase, TNF-α, IL-6, KC levels, and ICAM-1 and TLR-2 expression when compared to the same length of treatment to LPS or GLP alone, thus indicating the immunomodulatory and pro-inflammatory impacts of GLP.	(188)
Roundup (containing 0.2 g GLP/kg and 0.1 g POEA/kg), GLP (0.2 g/kg), POEA (0.1 g/kg), and a mixture of GLP (0.2 g/kg) + POEA (0.1 g/kg), applied to rats: immediate respiratory effects were more severe and more prolonged after POEA application than in the GLP group. All formulations caused diarrhea and blood-stained weeping (GLP caused transient diarrhea without nose bleeds), and the deaths were observed only from POEA; both POEA and GLP caused lung hemorrhages and lung epithelial cell damage.		(189)
*Hepatotoxicity*		
n. m.	GLP, applied at 5 and 50 mg/L to carps, caused oxidative stress, hepatic inflammatory response, and lipid metabolism disorder.	(196)
Roundup (44.1%, GLP), applied at 0.8503 mL/kg/day (375 mg/kg GLP) to rats, elevated AST, ALT, and MDA levels and apoptotic markers, caused hydropic swelling with nuclear pyknosis in the hepatocytes and degraded the cytoplasmic organelles.	n. m.	(197)
n. m.	GLP, applied at 0.05 and 0.5 μg/kg bw to lizards, caused suffering, severe hepatic condition, fibrotic formations, and xenoestrogenic oxidative stress.	(199)
n. m.	GLP, applied at 0.1, 0.5, 1.75, and 10 mg/kg bw to rats, caused dose-dependent weight decrease, oxidative stress, DNA damage in the liver cells and leukocytes, and inhibited AChE.	(200)
Roundup Grand Travaux Plus (no. 2020448, 450 g/L GLP), applied at 0.1 ppb or 50 ng/L GLP equivalent (daily intake of 4 ng/kg bw/day of GLP) to rats, altered the gene expression referred to mRNA splicing and small nucleolar RNA gene expression in liver and kidney, disrupted nucleolar structure in hepatocytes, upregulated genes controlling chromatin structure and downregulated the genes of the respiratory chain complex I and the tricarboxylic acid cycle mainly were downregulated, and modulated the mTOR and phosphatidylinositol signaling pathways; fibrosis, necrosis, phospholipidosis, mitochondrial membrane dysfunction and ischemia were also observed.	n. m.	(201)
Roundup Grand Travaux Plus (no. 2020448, 450 g/L GLP), applied at 0.1 ppb or 50 ng/L GLP equivalent (daily intake of 4 ng/kg bw/day of GLP) to rats, disturbed protein involved in organonitrogen metabolism and fatty acid β-oxidation that caused oxidative stress, peroxisomal proliferation, steatosis, necrosis, and hepatoxicity.	n. m.	(202)
MON 52276 and GLP, applied at 0.5, 50, 175 mg/kg bw/day of GLP equivalent to rats, caused ceca accumulation of shikimic acid and 3-dehydroshikimic acid, suggesting inhibition of 5-enolpyruvylshikimate-3-phosphate synthase of the shikimate pathway in the gut microbiome, and increased the cysteinylglycine, γ-glutamylglutamine, and valylglycine levels in the cecal microbiome; GLP dysregulated the nicotinamide, branched-chain amino acid, methionine, cysteine, and taurine metabolism, thus indicating oxidative stress, whereas MON 52276 had more pronounced effects than GLP on the serum metabolome; GLP and MON 52276 increased levels of *Eggerthella* spp., *S. zoogleoides*, *A. johnsonii*, and *A. muciniphila*, whereas *S. zoogleoides* was higher only with MON 52276 exposure.		(203)
*Hepato- and nephrotoxicity*		
MON 52276 (EU), MON 76473 (United Kingdom), MON 76207 (United States) and GLP, applied at 0.5, 50, and 175 mg/kg bw/day of GLP equivalent to rats; MON 52276 and MON 76473, but not GLP and MON 76207, caused oxidative stress and unfolded protein responses. MON 52276 but not GLP increased hepatic steatosis and necrosis, whereas MON 52276 and GLP altered the expression of genes in the liver, reflecting TP53 activation by DNA damage and circadian rhythm regulation; genes most affected in the liver were similarly altered in kidneys; in the liver, MON 52276 decreased miR-22 and miR-17, GLP decreased miR-30, whereas miR-10 levels were increased. MON 52276 and GLP altered methylation of CpG sites, and GLP increased apurinic/apyrimidinic DNA damage formation in the liver;		(214)
n. m.	GLP, applied to zebrafish alone and with heavy metals or metalloids at a 10-ppb dose, caused metal and GLP-metal mixture specific effects on kidney development displayed as alteration of *pax2a* and *kim1* genes expression and mitochondrial dysfunction.	(224)
Roundup (360 g/L of GLP) and GLP, applied to rats at 3.6, 50.4, and 248.4 mg/kg bw of GLP equivalent; Roundup altered levels of the kidney biomarker, oxidative stress markers and membrane-bound enzymes more profoundly than GLP alone; Roundup accumulated more than GLP residue in the kidney tissue and caused more lesions whereas GLP alone was not nephrotoxic to the renal function.		(226)
*Carcinogenicity*		
Roundup (48% w/v GLP, dosed at 269.9 mg/kg) and GLP (134.95 mg/kg), in male rats; Roundup induced the leakage of hepatic intracellular enzymes, ALT, AST and ALP, and time-dependent depletion of GSH levels and induction of hepatic oxidative stress; GLP increased NO levels more than Roundup after 2 weeks of treatment, and both herbicides increased TNF-α levels.		(240)
Roundup 360 Plus, GLP, and AMPA, applied at 1–1000 μM, caused DNA damage in PBMCs and increased ROS; GBH was harmful at 5 μM, while GLP and AMPA were toxic at 250 μM and 500 μM, respectively.		(241)
Roundup (>41% GLP-IPA), applied at 25 and 50 mg/kg bw to mice, increased CAs and MN induction and decreased mitotic index indicating cytogenetic and chromosomal damage.	n. m.	(242)
n. m.	GLP, applied at 0.5, 2.91, and 3.5 μg/mL to hepatic cells, caused dose-dependent primary DNA damage and oxidative stress.	(244)
n. m.	GLP, applied at 0.5, 0.1, 0.05, 0.025, and 0.0125 μg/mL, to human lymphocytes; effects: chromosomal aberration and an increase in micronuclei frequencies significantly increased at all tested concentrations, with exception of 0.0125 μg/mL; only 0.5 μg/mL GLP increased the frequency of nucleoplasmic bridges.	(245)

a n. m.: not measured.

**Table 3:** Clinical Studies on GLP and GBH Toxicity^a^

GBH	GLP	ref.
*Endocrine and reproductive system diseases*		
Roundup use was overrepresented in the adverse birth and developmental effect group of 6 of 14 children (43%) who had parent-reported ADD/ADHD.	n. m.	(121)
The Ontario Farm Family Health Study, executed on 3984 pregnancies, showed no associations between miscarriage, preterm delivery and small-for-gestational-age births or altered sex ratio and overall farm activities; however, increased risk of reproductivity issues was associated with the combined use of farm activities and mixing various pesticides, including atrazine, GLP, organophosphates, 4-[2,4-dichlorophenoxy] butyric acid, and insecticides.		(126)
The Ontario Farm Family Health Study, that analyzed 2012 pregnancies, showed no strong or consistent associations between pesticides (including GLP) exposure and TTP, however GLP (among 6 of 13 pesticides) exposure was associated with a decrease in fecundability (conditional fecundability OR range = 0.51–0.80).		(127)
In the Ontario Farm Family Health Study, a total of 2110 women provided information on 3936 pregnancies, including 395 spontaneous abortions; results: moderate increases in risk of early abortions for preconception exposures to phenoxy acetic acid herbicides [OD = 1.5; 95% confidence interval (CI), 1.1–2.1], triazines (OR = 1.4; 95% CI, 1.0–2.0), and any herbicide (OR = 1.4; 95% CI, 1.1–1.9); for late abortions, preconception exposure to GLP (OR = 1.7; 95% CI, 1.0–2.9), thiocarbamates (OR = 1.8; 95% CI, 1.1–3.0), and the miscellaneous class of pesticides (OR = 1.5; 95% CI, 1.0–2.4) was associated with elevated risks.		(128)
A retrospective study of TTP of 2592 Colombian women exposed to GLP/GBH, sprayed on cocaine and poppy farmlands for illicit crop eradication, revealed differences between women from Boyaca (non-GLP exposed) and Putumayo and Narino (illicit crops and intensive GLP-based eradication spray program), and Valle del Cauca (a sugar cane region with over 30 years-long use of GLP and others chemicals), where the risk of longer TTP was the highest (fecundability OR 0.15, 95% CI 0.12, 0.18).		(129)
n. m.	A mean urinary GLP level of 3.40 ng/mL (range 0.5–7.20 ng/mL, LOD of 0.1 ng/mL) was detected in 71 women with singleton pregnancies in Central Indiana; higher GLY levels were found in the women living in rural areas (*p* = 0.02), and in those who consumed caffeinated beverages however none of the drinking water samples had detectable GLP levels; in 93% of the women, the GLP level was higher than the LOD; there were no correlations between the GLP levels and fetal growth indicators such as birth weight percentile and head circumference; however, higher GLP urine levels were significantly correlated with shortened gestational lengths.	(130)
According to a case-referent study with 261 matched pairs executed in Comunidad Valenciana, Spain, the paternal occupational exposure to some pesticides (including glufosinate, i.e., GLP’s metabolite) was associated with increased, yet not statistically significant risk (adjusted OR 2.45, 95% CI 0.78–7.70) of congenital malformations.	n. m.	(131)
In the California Birth Defects Monitoring Program, the maternal residential proximity within 1000 m of pesticide applications was associated with NTDs, anencephaly, and spina bifida; 35.2% of mothers of cases and 26.8% of percent of mothers of controls; among other pesticides, GLP exposure was associated with an elevated risk of NTD-type of congenital malformations.		(132)
*Cardiovascular diseases*		
The 153 patients, poisoned with acute GLP-SH ingestions, displayed prolonged QTc interval followed by intraventricular conduction delay and first-degree atrioventricular block; a more prolonged QTc interval was observed in nonsurvivors than in the survivors; a significantly increased risk of death was associated with the corrected QT interval and age.	n. m.	(159)
The 232 patients (29 dead), intoxicated with GLP-SH, displayed an increased level of lactate [6.5 ± 3.1 mmol/L in nonsurvivors, 3.3 ± 2.2 mmol/L in survivors], which was associated with 30-day mortality; besides lactate, age >59 years, corrected QT interval >495 ms and potassium >5.5 mmol/L were independent risk factors for 30-day mortality.	n. m.	(160)
50% GLP-concentrated Roundup nonintentional ingestion by a 30-year-old woman caused syncope in the setting of ECG findings of a LBBB evolving into a type I Brugada pattern.	n. m.	(161)
Case 1: Suicidal mouthful ingestion of Roundup (360 g/L GLP) by a 69-year-old man caused a red throat and furred tongue without pharyngeal edema, as well as erythema in the mucous membrane of arytenoids.	n. m.	(162)
Case 2: A 44-year-old man presented vomiting 30 min after having ingested Pistol EV (250 g/L GLP and 40 g/L and diflufenicanil), which caused mild bilateral mydriasis, a dysrhythmia, altered consciousness, hypotonia, tachycardia, severe metabolic acidosis and a marked hyperkalaemia, that finally resulted in a lethal cardiovascular arrest. The toxicity is almost related to GLP since diflufenicanil is not toxic: DL50 in rats >2500 mg/kg.		
Case 3: A 51-year-old man, who attempted suicide by ingesting 2 large glasses of Roundup (360 g/L GLP), displayed circulatory shock, metabolic acidosis, respiratory distress and and disseminated intravascular coagulation, that resulted in a hemodynamic disturbance and a lethal multiple organ failure.		
Case 4: A 64-year-old woman, who ingested a glass of Roundup (360 g/L GLP), presented respiratory distress and hypersialorrhea followed by cardiac shock, dysrhythmia caused by hyperkalaemia, metabolic acidosis, erosion of the digestive tract and hepatic toxicity.		
Case 5: A suicidal attempt by ingesting a glass of Glyper (360 g/L GLP) by a 46-year-old woman caused blood vomiting and diarrhea followed by a sore throat, dysphonia, metabolic acidosis, sinusal tachycardia and dispersed pain.		
Case 6: In a 60-year-old-man, self-poisoning by ingesting Roundup (360 g/L GLP) or Grivolax (paraquat) caused shock, sweating, pulmonary obstruction, an impaired renal function, metabolic acidosis, and hemodynamic disturbance.		
Case 12: A 59-year-old man, who voluntarily drank Verdys (360 g/L GLP) and Decis (deltamethrin), displayed a Glasgow coma scale of 15 and metabolic acidosis with high lactate (4.5 mmol/L).		
Case 13: A 65-year-old man, who accidentally ingested a mouthful of GBH, presented sore throat and dysphagia without ulceration.		
GLP-Trimesium (Touchdown, ∼ 150 mL) caused rapid deaths of a 6-year-old boy and a 34-year-old after accidental and intentional ingestion, respectively; the edema of the mucus membranes of the airways, erosion of the mucus membranes of the GIT, pulmonary edema, cerebral edema, and dilated right atrium and ventricle of the heart were reported.	n. m.	(163)
A suicidal ingestion of ∼400 mL of nian–nian-chun (Chinese GLP-SH containing 41% GLP-IPA) was fatal to a 57-year-old woman; the intoxication caused drowsy consciousness, metabolic acidosis, ventricular tachycardia, refractory respiratory failure, oral ulcers, blood-tinged saliva, crackles on chest auscultation, cold extremities, and refractory shock resulting in the death.	n. m.	(164)
Accidental ingestion of 100 mL GLP-SH by a 65-year-old woman caused severe throat soreness, hypoxemia, hyperkalemia, and hypotension, followed by acidosis, pulmonary edema and acute kidney injury, aspiration pneumonitis, and the intestine ileus of the intestine.	n. m.	(165)
Ingestion of ∼100 mL of GLP-SH by a 47-year-old man caused mildly decreased consciousness, cardiopulmonary failure, persistent ventricular tachycardia, profound shock refractory to inotropic agents, and metabolic acidosis.	n. m.	(166)
GLP-SH poisoning caused circulatory shock and unconsciousness in a 52-year-old man	n. m.	(167)
*Pancreas diseases*		
Ingestion of 75 mL of Glycel herbicide (40.6% GLP-SH) along with 120 mL of alcohol caused sweats, epigastric tenderness on palpation of the abdomen, buccal and posterior pharyngeal mucosa showed congestion and ulceration, and vomits in a 35-year-old male patient.	n. m.	(190)
Suicidal ingestion of ∼100 mL GLP-SH caused irritation, chemical pneumonitis, bilateral crackles, epigastric tenderness, respiratory failure, and acute pancreatitis in an 89-year-old man.	n. m.	(191)
*Larynx diseases*		
The 36 cases of laryngeal injury were found in the 1992–1996 survey including 53 cases of GLP-SH intoxications; elevated blood WBC counts and longer hospitalization were reported for the patients with laryngeal injury when compared with patients with no laryngeal injury; the laryngeal injury was correlated with aspiration pneumonitis.	n. m.	(192)
Suicidal ingestion of 250 mL of GLP-SH by a 52-year-old woman caused slight aspiration pneumonitis and the intestinal ileus, upper-airway obstruction, and hyperplasias.	n. m.	(193)
*Liver diseases*		
n. m.	The examination of urine samples of NAFLD/NASH patients, hospitalized at the University of California at San Diego NAFLD Research Center, revealed GLP levels of 0.373 μg/L in women and 0.215 μg/L in men, GLP residue levels of 833 μg/L in women and 0.594 μg/L in men; in multivariate models adjusting for age, sex, and BMI, as compared to patients without NASH, AMPA and GLP residue were elevated in patients with definite NASH; patients with advanced fibrosis had, respectively, elevated AMPA (0.196 μg/L vs 0.365 μg/L), GLP residue (0.525 μg/L vs 0.938 μg/L), and GLP (0.230 μg/L vs 0.351 μg/L).	(207)
*Kidneys diseases*		
n. m.	A case-control study executed in Padavi-Sripura hospital in Trincomalee district among CKD patients (180 control, 125 cases; 107 cases were farmers from paddy fields), revealed that the highest risk for CKD was observed among participants exposed to well water (OR 2.52, 95% CI 1.12–5.70) and drinking water from an abandoned well (OR 5.43, 95% CI 2.88–10.26) and GLP pesticide sprays (OR 5.12, 95% CI 2.33–11.26); water analysis confirmed the GLP presence levels (1 μg/L) in the abandoned wells; a significantly higher risk was observed of CKD for male farmers with OR 4.69 (95% CI 1.06–20.69) in comparison to their female counterparts.	(216)
n. m.	Analysis of urine samples of 10 Sri Lankan agricultural nephropathy patients and from two sets of controls, one from healthy participants (*N* = 10) from the same endemic area and the other from a nonendemic area (*N* = 10; Colombo district) confirmed the elevated GLP levels in the patients from endemic areas, with the highest urinary GLP concentration recorded in SAN patients (range 61.0–195.1 μg/g creatinine).	(217)
n. m.	GLP (270–690 μg/kg) and AMPA (2–8 μg/kg) were detected in all topsoil samples collected from agricultural fields, water samples from nearby shallow wells and lakes, and sediment samples from lakes, which was associated with CKD among Roundup-using farmers in Sri Lanka; GLP displayed a strong and moderate positive linear relationship with amorphous iron oxides and organic matters; in lakes, the GLP levels were between 28 to 45 μg/L, whereas no AMPA was detected; in all groundwater samples, 1–4 μg/L GLP was found, whereas 2–11 μg/L AMPA was detected only in four out of nine samples; in all sediment samples, 85–1000 μg/kg GLP was found, displaying and a strong linear relationship with the organic matter content, whereas 1–15 μg/kg AMPA in seven out of nine sediment samples.	(218)
Among 526 workers (cases), occupationally exposed to GLP/GBH and another 442 nonexposed administrative staffs (controls); the concentration level of glyphosate in the air of workshop was detected <0.03–48.91 mg/m^3^ and positively correlated with hepatorenal abnormalities in the case group.		(225)
n. m.	GLP at 0.278 ± 0.228 μg/mL was identified among 11.1% of children of the epidemiological study, however any GLP impact on the levels of kidney injury biomarkers, including ACR, NGAL, and KIM-1 was excluded.	(227)
*Cancer*		
In the AHS, a prospective cohort study of licensed pesticide applicators from North Carolina and Iowa, conducted among 54 251 applicators, 44 932 (82.8%) used GLP/GBH, including 5779 incident cancer cases (79.3% of all cases), no association was apparent between GLP/GHB and any solid tumors or lymphoid malignancies overall, including NHL and its subtypes. There was an increased risk of AML among the highest exposed group, compared with never-users, however this association was not statistically significant and required further confirmation.		(236)
Occupational exposure to herbicides (GLP, phosphonoglycine) and fungicides (mancozeb, maneb, zineb, ziram, benzimidazole, and fosethyl-aluminum) was associated with a high risk of cutaneous melanoma (OD 2.58; 95% CI 1.18–5.65) among Italian and Brazilian subjects (399 cases of 800). The 47 subjects were exposed at an occupational level at pesticide factories. Additional sun exposure increased the risk (OD 4.68; 95% CI 1.29–17.0).		(246)
*Multiorgan toxicity*		
A 75-year-old man, who intentionally digested >150 mL of Roundup GLP-SH in a suicidal attempt, presented impaired consciousness (Glasgow Coma Scale 6), decreased mean arterial blood pressure, severe cardiovascular instability, and acute respiratory distress syndrome, followed by fever, abdominal and transverse colon distention and, diarrheas, reduced bowel sounds, and abundant erosions, abscess formation, necrosis of perienteric fat tissue, and fibroblastic reaction without evidence of mucosal ischemia.	n. m.	(209)
In the prospective observational case series conducted in Sri Lanka among 601 patients, exposed to GBH (in the majority 36% w/v-concentrated GLP), 27.6% of the patients were asymptomatic, 64% had minor poisoning and 5.5% of patients had moderate to severe poisoning; there were 19 deaths (case fatality 3.2%) with a median time to death of 20 h; in fatal cases, gastrointestinal symptoms, respiratory distress, hypotension, altered level of consciousness and oliguria were observed; death was strongly associated with greater age, larger ingestions and high plasma GLP concentrations on admission (>734 μg/mL) and the apparent elimination half-life of GLP was 3.1 h (95% CI 2.7 to 3.6 h).		(249)
A 56-year-old woman, who ingested ∼500 mL GLP-IPA herbicide, presented hypotension, vigil coma, hyperkaliemia, respiratory, renal failure, and extensive bilateral ischemic lesions of the brain stem white matter and pons.	n. m.	(250)
A 67-year-old male, who attempted suicide by ingesting 250 mL of Touchdown IQ (44.75% GLP salt), displayed mild upper abdominal discomfort, nausea, vomiting, metabolic acidosis, hypoxemia, and hyperkalemia resulting in atrial fibrillation with tall T waves.	n. m.	(251)
Ingesting commercial GBHs, including Roundup Maxload, by a 65-year-old female caused consciousness depression, blood pressure and respiratory rate drops, metabolic acidosis, and extreme hyperkalemia.	n. m.	(252)
A 65-year-old woman with a history of breast cancer (at 62 years), depression, and breast cancer recurrence (at 65 years), was unconscious after a suicidal attempt by ingesting ∼500 mL GlyphoAce 41% GLP salt; the diagnosis included severe hyperkalemia and renal dysfunction, abdominal distention and tenderness without peritoneal signs, respiratory issues, paralytic ileus and bowel wall edema and massive amounts of fluid inside the intestine.		(253)

a n. m.: not measured.

## 3 Glyphosate Toxicity to the Liver, Kidneys, and Endocrine, Reproductive, and Cardiopulmonary Systems

### 3.1 GLP Impact on Endocrine and Reproductive Systems

#### 3.1.1 Endocrine System and Endocrine-System Disrupting Chemicals

**Figure 1:**
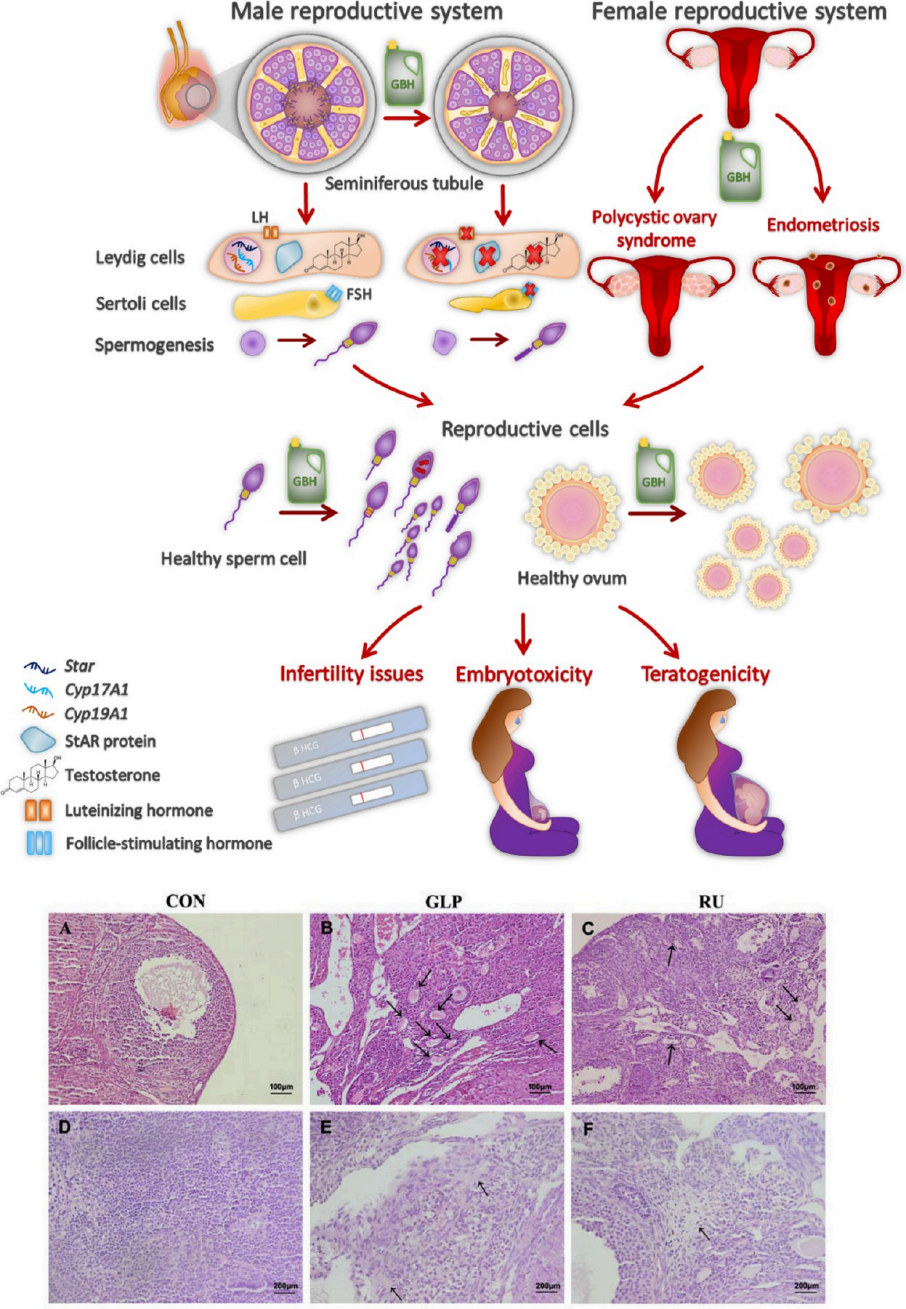
The GBH impact on endocrine and reproductive systems. The scheme presents an overview of GLP and GBH toxicity in male and female reproductive systems. By deviating the hormone production and activities, the herbicides impact the development and functionality of Sertoli cells, Leydig cells, and sperm cells in seminiferous tubules of the testes and oocytes in ovaries. Besides, they cause, e.g., endometriosis and polycystic syndrome, thus leading to infertility, embryotoxicity, and teratogenicity. (A–F) Hematoxylin and eosin (X 100) staining of ovarian sections of GLP-treated mice showing (A) the normal histological ovarian follicle structure and (B, C) increased numbers of atretic follicles after mice treatment with (B) GLP and (C) Roundup. (D–F) Hematoxylin and eosin (X 200) staining showing (D) the normal ovarian interstitial cell structure and (E, F) the interstitial fibrosis after mice treatment with (E) GLP and (F) Roundup. Black arrows indicate lesioned regions. Adapted with permission from ref (37). Copyright Elsevier 2018.

The endocrine system regulates metabolism, respiration, mood, mechanosensory perception and movement, growth, reproduction, sexual development by producing and secreting hormones^56−58^ (Figure 1). Endocrinology mentions two categories of endocrine system diseases, namely, (i) hormone imbalance, resulting from the failure of the endocrine feedback system and causing hyposecretion or hypersecretion, i.e., hormone deficiency or hormone excess, respectively, and (ii) diseases resulting from infections, injuries, tumors, or genetic issues, which may lead to hormone imbalance. Major endocrine system diseases involve diabetes, hyper- or hypothyroidism, adrenal insufficiency, Cushing’s disease, and sex hormone disorders, including hermaphroditism, hypogonadism, precocious puberty, and multiple endocrine neoplasia.^56−59^ Exposure to environmental toxicants and EDCs also dysregulates the hormonal balance homeostasis.^60,61^ The EDCs, first reported in the 1990s, include pharmaceuticals, plastics (phthalates), pesticides, cosmetics, detergents, and phytoestrogens. Experimental pieces of evidence highlight the endocrine-disrupting activity of GLP and GBHs.^31,35,52^ Nonetheless, according to the EPA’s EDSP and EFSA, there is no sufficient evidence to support the endocrine-disrupting effects of GLP, as it exhibits no direct interaction with the EAT pathways.^35,52^ However, this issue has still been debated in the EU and Brazil.^62^

Ten key characteristics (KCs) of the EDCs have been classified to describe their mechanistic impacts on the endocrine system functionality.^63^ (i) The EDCs interact or activate hormone receptors, e.g., androgen receptor (AR), estrogen receptors (ERα, ERβ), and progesterone receptor (ProgR). As revealed by preclinical studies, GLP plays an ambiguous role as a hormone mimic,^64,65^ being either antiestrogenic in hormone- and dose-dependent manner activity^41^ or ER-antagonistic^66^ in a ligand-independent and GLP-specific manner.^67^ Contrariwise, (ii) as an EDC, GLP may antagonize hormone receptors. However, no clear evidence for the GLP-mediated antagonistic effect on hormone activity has so far been reported.^41,66^ For instance, using liver cells (HepG2), genotoxic, antiestrogenic, and aromatase-disruptive activities of GLP were compared with these of Roundup Express, Bioforce (Extra 360), Grands Travaux 400, and Grands Travaux 450. Among these formulations, applied in subagricultural dilutions (0.5–5 ppm), the toxicity of pure GLP was the lowest or negligible, whereas the carcinogen, mutagen, and reprotoxic actions of these formulations depended on the GBH’s adjuvant content rather than on the GLP concentration.^41^ These outcomes are, however, in contrast to other pesticides.^68^ To continue, (iii) GLP regulates the gene expression of hormone receptors in a dose-dependent manner in hormone-dependent cancer cells.^66^ However, another study excluded this activity in Leydig cells.^42^ Yet, studies on pre- and postnatal,^47^ perinatal,^69^ and prepubertal rats^70^ provide no support for this conclusion. Moreover, (iv) GLP alters the signal transduction, cell cycle, and cellular growth in hormone-responsive cells without direct interaction with the hormone receptor,^32,71^ as revealed by a study on prepubertal rat-derived Sertoli cells.^12^ Consecutively, (v) GLP and GBH induce epigenetic modifications in hormone-producing or hormone-responsive cells. Examples of the in vivo GLP hazardous epigenotoxicity include epimutations (epigenetic traits), DNA hypomethylation of oncogenes,^72^ histone targeting and chromatin remodeling,^69^ dose-dependent hypermethylation of the CpG islands of the ER gene promoters,^73^ transgenerational (F1, F2, and F3) pathologies,^43^ miRNA and circRNA dysregulation-associated metabolic and neurodevelopmental disorders (NDDs),^74,75^ and organ malformations and congenital anomalies.^76^ Moreover, (vi) GLP and GBHs alter steroidogenesis and hormone synthesis by disrupting the expression of steroidogenic acute regulatory (StAR) protein,^77−81^ demonstrated in vivo.^40−42^ Importantly, in a study on human placental JEG cells, toxic and inhibitory activities of Roundup, dosed at the agricultural concentration, were superior to those of GLP.^38^ Furthermore, (vii) GLP and GBHs interfere with hormonal balance throughout the body by altering hormone transport across endocrine cell membranes or vesicle secretion.^31^ Roundup indirectly influenced the plasma membrane-linked endocrine disruption in pregnant female rats,^82^ male pubertal rats,^83,84^ and perinatal mice^37^ (Figure 1A–1F). However, contradictory data have also been presented.^42,70,85,86^ In addition, (ix) as EDC, the GLP and GBHs are expected to deviate from hormonal metabolism and clearance mechanisms, including first-pass metabolism in the liver and excretion in the kidney. Yet, no experimental data have confirmed this mechanism.^31^ Finally, (x) GLP and GBHs influence the fate of hormone-producing or hormone-responsive cells by direct or indirect changes in differentiation, proliferation, apoptosis, DNA repair, hypoxia, mutagenesis, and migration of target of effector endocrine cells.^32,42,66,67,71,87−89^

#### 3.1.2 Endocrine System Disruptors of the Hypothalamus–Pituitary–Peripheric Glands Axes

The hypothalamus coordinates the endocrine system. By consolidating signals from upper cortical inputs, autonomic function and physical cues, and peripheral hormonal feedback, the hypothalamus provides specific signal outputs to the pituitary gland that subsequently supplies the endocrine system with hormones stimulating the peripheral glands.^90^ As EDCs, GLP and GBHs dysregulate the functionality of the hypothalamus-pituitary and its connections with HPP glands axes, including adrenal (HPA), thyroid (HPTh), and gonadal axes, i.e., ovaries (HPO) and testes (HPT).^35^ However, the EPA, EFSA, and the Organization of Economic Co-operation and Development (OECD) have recently questioned the endocrine-disrupting activity of GLP and excluded GLP as an EDC. A comprehensive experimental review conducted within EPA’s EDSP and the European Centre for Ecotoxicology and Toxicology of Chemicals (ECETOC) critically verified an endocrine-modulating or adverse potential of GLP on steroidogenesis and the EAT pathways in humans, other mammals, and wildlife.^52,91^ Contemporary contradictory reports highlight, though, the harmful GLP impact on steroidogenesis, gonadal, and thyrotropic axes, and the reproductive system.^23,30,33,37,43,49,82^

For example, GBH induced dysregulation of the HPTh axis, causing osteoporosis, skeletal dysfunctionality, and hypothyroidism in Kalach 360 SL-fed rats female and offspring. Malfunctions in the osteocytes and thyroid cells’ activity altered the estrogen, calcium, phosphates, phosphatase alkaline, and vitamin D levels, as well as decreased triiodothyronine and thyroxine levels, associated with an increased plasma level of thyroid-stimulating hormone. These malfunctions led to subosteoporotic thinning and discontinuity of bone trabecular with a significant decrease in intertrabecular links.^92^ The impact of excessive exposure to GLP or GBHs on the functionality of the HPTh axis was summarized elsewhere.^52^ Examples of the impacts of these herbicides on the HPA, HPO, and HPT axes are discussed below.

#### 3.1.3 Preclinical Studies of GLP Impact on the Endocrine and Reproductive Systems

##### 3.1.3.1 Reproductive System Diseases

Reproductive system diseases, also called generational pathologies, comprise (i) genetic and congenital abnormalities, including epimutations and epigenetic fertility issues, (ii) functional or structural genital disorders associated with disruption of the endocrine system and hormonal disorders, (iii) disturbances of pregnancy and embryonal or fetal development, and parturition, (iv) infections, and (v) tumors.^93,94^ The pesticides and herbicides belong to well-known toxins causing menstrual cycle disturbances, infertility, subfertility, prolonged time-to-pregnancy (TTP), spontaneous abortion, miscarriage, stillbirth, hemangioma birthmarks, congenital malformations in the offspring, as well as endocrine and hormonal issues, and musculoskeletal and neurobehavioral disorders (NBDs).^95,96^

##### 3.1.3.2 Epigenetics

Epigenetics is a branch of genetics treating the inheritance of stable phenotype changes that arise from affected gene activity or expression without alterations in the DNA sequence, manifested as epigenetic traits (epimutations) in a chromosome that result from environmental (extracellular) impacts on the DNA methylation, chromatin remodeling, and transgenerational epigenetic inheritance, etc.^97^ GLP and GBHs trigger epimutations. For instance, the ancestral environmental exposure of F0 female rats to GLP caused no or minor epigenotoxicity in the F0 and F1 generations but became severely toxic in the F2 and F3 offspring. The transgenerational pathology, including differential DNA methylation regions, caused prostate disease, obesity, kidney disease, ovarian disease, and parturition abnormalities.^43^ Organ malformations and GLP tissue residuals, putatively associated with congenital anomalies, were observed in one-day-old piglets born by females exposed to GLP in the first 40 days of pregnancy. The organs most severely damaged in the piglets were the lungs, liver, kidney, brain, gut wall, and heart, whereby the highest GLP tissue concentration, quantified by enzyme-linked immunosorbent assay (ELISA), was in the lungs and hearts, whereas the lowest was in muscles.^76^

##### 3.1.3.3 Steroidogenesis and Gonads

GLP and GBHs destroy the production and functionality of gonads (Figure 1A). Roundup attenuated progressive motility and destroyed the mitochondrial integrity of human sperm,^44^ whereas GLP alone decreased the sperm’s motility and caused sperm DNA fragmentation.^45^ Moreover, GLP negatively affected sperm mitochondrial respiration efficiency and worsened the harmful effect of dihydroxytestosterone on sperm mitochondria.^98^ Agent-specific impacts were evaluated, as well. In pigs, both herbicides caused dose-dependent decreases in sperm motility, viability, mitochondrial activity, and acrosome integrity but no changes in the DNA structure were observed. However, the toxicity of Roundup was more profound than that of GLP alone.^11,15^

GLP and GBH affect signaling pathways in cells responsible for adrenal gland steroidogenesis. The adult male rats’ exposure to Roundup triggered apoptosis, reduced systemic levels of corticosterone and adrenocorticotropic hormone receptors, and altered the level of StAR protein phosphorylation. The serum concentration of testosterone was decreased, as well as aromatase levels and luteinizing hormone (LH) and follicle-stimulating hormone (FSH) gonadotropins deviated in male rat offspring of the perinatally GLP-exposed females.^99^ GLP and Roundup endocrine cytotoxicity were evaluated in male estuarine crabs (*Neohelice granulate*). Both herbicides decreased sperm count in spermatophores from the vas deferens and inhibited the secretion and/or transduction of the androgenic gland hormone, thus dysregulating spermatogenesis.^100^ Neuroendocrine and immune toxicity of GLP was demonstrated in lizards (*Salvator merianae*). Blood morphology of the GLP-treated lizards revealed an elevated level of plasma corticosterone, decreases in the total white blood cell count and natural antibodies titres, and an increase in the lobularity index, thus indicating immunosuppression and symptoms of chronic infection, although differential white blood cell count, heterophils/lymphocytes index, and complement system have not deviated.^101^

##### 3.1.3.4 Testes

GBHs affect testes development, leading to changes in testosterone levels, seminiferous tubules, and puberty progression (Figure 1A). In prepubertal rats, Roundup decreased testosterone levels without affecting corticosterone or estradiol levels, and it altered seminiferous tubules and germinal epithelium in a dose-dependent manner.^84^ Feeding prepubertal male rat offspring GLP-containing soy milk had toxic effects. GLP reduced testosterone levels and Sertoli cell numbers and increased the percentage of degenerated Sertoli and Leydig cells. Additionally, it reduced spermatid numbers, increased epididymal tail mass, and decreased seminiferous tubule diameter.^102^ Perinatal mouse exposure to GLP or Roundup at acceptable daily intake concentrations in drinking water had agent-specific outcomes. GLP, but not Roundup, deviated from testis morphology, decreased testosterone serum levels, and reduced undifferentiated spermatogonia numbers by 60% in the GLP group. It was associated with the downregulation of the *Sal4* gene and the up-regulation of the *Nano3* gene related to germ cell differentation, as well as the *Bax* and *Bcl2* genes, involved in apoptosis.^34^ Moreover, maternal gestational exposure to Roundup altered masculinization of male offspring masculinization. At 60 days old, males from Roundup-treated dams showed increased sexual partner preference scores, elevated serum testosterone and estradiol levels, LH and FSH mRNA expression, LH and FSH gonadotropin protein content in the pituitary gland, deviated sperm production, and testicular morphology alterations. They also experienced an early onset of puberty.^99^ Similar outcomes were observed in a study on attenuating the effects of Roundup on male mouse offspring from females exposed to Roundup in drinking water from the fourth day of pregnancy to the end of the lactation period. In F1 males from the GBH group, testicular descent was delayed, spermatozoa in the cauda epididymis were reduced, seminiferous epithelium height was decreased, intratesticular testosterone levels were increased, and the HPT axis was dysregulated.^33^

Exposure of both prepubertal and postpubertal male rats to GBHs promotes mammary gland development by increasing collagen fiber organization and terminal end buds. Additionally, GBH-treated rats exhibited higher levels of mast cell infiltration, ERα expression, and proliferation index than control rats.^70^ Roundup induced Ca2+-dependent oxidative stress and activated multiple endoplasmic reticulum stress-response pathways, leading to Sertoli cell death and reduced spermatogenesis in prepubertal rat testes. Exposure to GLP alone produced similar effects.^12^ A study comparing GLP, POEA, and GBHs (Roundup and Glyphogan) at concentrations ranging from environmental- to agricultural-use levels in an immature Sertoli cell line (TM4) revealed that the GBH formulation caused mitochondrial dysfunction, disrupted cellular detoxification systems, and led to lipid droplet accumulation and necrosis. Overexposure to POEA resulted in excessive lipid accumulation, suggesting that cell death followed immediate penetration and overload of the formulants inside the cells.^103^ Contradictory results emerged from comparing the GBH formulation (Glyfonova) and an equivalent amount of GLP on rat testes and androgen functionality. GLP had no significant impact on testes or testosterone synthesis, whereas Glyfonova only slightly upregulated the steroidogenic genes Cyp11a1 and Cyp17a1, related to aromatase (Figure 1A).^104^

Perinatal exposure of rats to Roundup Transorb during a critical period of sexual differentiation led to HPT axis dysfunction, including increased LH and FSH mRNA expression levels, elevated LH protein in the pituitary gland, higher serum LH concentrations in adult male offspring, and subsequent pro-angiogenic effects. This dysregulation boosted blood testosterone levels, enhanced sperm production, and increased the weight of reproductive organs.^99^ In rats exposed to Roundup in utero and postnatally, there was documented evidence of an increase in anogenital distance.^30^ Meanwhile, exposure of prepubertal rats to Roundup resulted in an antiandrogenic effect, lowering systemic testosterone levels and inhibiting male puberty entry.^84^ Male mice exposed to Roundup during gestation and lactation experienced delayed testis descent and decreased spermatozoa in the cauda epididymis.^33^ Lastly, when adult rats were orally administered technical-grade Roundup, it disrupted the transcription of StAR mRNAs, leading to lipid droplet accumulation in the adrenal gland, increased gland weight, and reduced levels of corticosterone, adrenocorticosterone, and phosphorylated CREB.^79^

##### 3.1.3.5 Ovaries

Treatment with GLP or GBH deviates from the functionality of the HP-ovaries axis, thus triggering ovarian failure and deteriorating the quality of oocytes (Figure 1). In mice, pure GLP dysregulated metaphase II oocyte quality, disrupted the microtubule organizing center, formation of a spindle fiber, and chromosomal alignment, and chelated zinc cations, which decreased its intracellular content and caused reactive oxygen species (ROS)-mediated embryo damage.^105^ A comparison of GLP and Roundup activities in pig oocytes revealed that Roundup impaired oocyte development and blastocyst rate deviated steroidogenesis in cumulus cells and increased intracellular levels of ROS, wherein the Roundup impact was higher than that of an equivalent amount of pure GLP.^106^ Disrupting activity of orally administered technical grade GLP and Roundup on ovaries was demonstrated in pregnant mice and their fetuses during the gestation period (first 19 days). The body, ovaries, liver weight, and mature follicles in treated mice decreased, whereas atretic follicles and interstitial fibrosis increased. Both progesterone and estrogen levels were significantly changed, as well as the expression levels of *GnRH* (gonadotropin-releasing hormone), *LHR*, *FSH*, *3β-HSD*, and *Cyp19a1* genes at the hypothalamic-pituitary-ovarian (HPO) axis. The herbicide treatment induced oxidative stress, manifested by increased T-AOC, CAT, and glutathione peroxidase (GSH-Px) activity and high malondialdehyde (MDA) content in the serum and ovaries. Finally, prenatal exposure to GLP altered the sex ratio of the litter^37^ (Figure 1A–1F).

##### 3.1.3.6 Uterus

Extensive studies on rats have demonstrated that excessive exposure to GLP or GBH destroyed the uterus’s development and functionality, as well as morphological and physiological features^46−49^ (Figure 1). For example, in adult ovariectomized rats subcutaneously injected with a GBH formulation, there were no changes in uterine weight or epithelial proliferation, but the GBH injection increased the luminal epithelial cell height and downregulated the ERα mRNA and protein levels in luminal epithelial cells, whereas the ERα was upregulated in the stroma. Moreover, the GBH injection upregulated ERβ and ProgR expression levels.^107^ In another study, the GBH exposure deviated the activity of ERα, ProgR, homeobox protein Hox-A10 (HOXA10), and Wnt7a that regulate uterine organogenetic differentiation, causing luminal epithelial hyperplasia and increases in the stromal and myometrial thickness.^108^ Ovarian follicular dynamics, associated with increased proliferation of granulosa and theca cells, was altered, expression of FSHR and GDF9 mRNA was downregulated, and proliferative activity of the uteri cells was decreased in GBH-exposed prepubertal lambs. Noteworthy, none of these outcomes were in the lambs treated with GLP or AMPA.^109^

Early postnatal exposure to GBH induced lasting morphological changes in the female rat mammary gland, including a fibroblastic-like stroma, a higher percentage of hyperplastic ducts, and increased expression of steroid hormone receptors^110^ (Figure 1). In prepubertal rats, GBH increased uterine sensitivity to estradiol, leading to endometrial hyperplasia characterized by increased luminal and glandular epithelial height and stromal nuclei density.^46^ In neonatal rats exposed to GBH, alterations in endometrial decidualization at implantation sites were associated with dysregulated expression levels of estrogen and progesterone receptors (ER and ProgR), as well as endocrine pathway-regulating markers (HOXA10) and proliferation markers.^47,48^ In another study involving rat females exposed to either pure GLP or GBH from gestational day 9 until weaning, herbicide exposure induced preimplantation losses in the F1 generation, increased 17β-estradiol serum levels, and upregulated ERα expression. GLP specifically downregulated ProgR mRNA expression. Additionally, HOXA10 and Lif genes were downregulated in herbicide-treated rats.^49^ In weaned pigs, GLP and Roundup administered through feed had insignificant effects on the vulvar size and the index of reproductive organs. However, they altered the uterine and ovarian ultrastructure and disrupted the synthesis and secretion of LH, FSH, GnRH, and testosterone. Roundup also caused an imbalance in hydrogen peroxide and MDA levels in reproductive organs^23^ (Figure 1). In cows, GLP directly stimulated estradiol secretion from granulosa cells, while both GLP and Roundup had varying effects on oxytocin and progesterone secretion from luteal cells, leading to deviations in the estrous cycle and uterine contractions that could result in infertility. Additionally, both formulations decreased prostaglandin secretion from endometrial cells but did not directly affect the basal and oxytocin-stimulated force of the motor functions of the myometrium.^111^

##### 3.1.3.7 Embryo- and Teratogenicity

GLP and GBH directly impact embryonic and fetal development, as evidenced by various experimental findings^112^ (Figure 1). For instance, GLP led to carbonic anhydrase inhibition and ROS-triggered cellular apoptosis in zebrafish embryos, resulting in multiorgan and body malformations.^113^ Lizards treated with Roundup and Panzer Gold formulations at different stages of embryonic development (3–5 and 33 days) exhibited embryonic and hematological alterations in their blood samples.^114^ Embryotoxicity and teratogenicity in *Xenopus laevis*, three GBH formulations (Roundup, Kilo Max, and Enviro Glyphosate) were higher than those of GLP alone. These GBHs caused cardiac and abdominal edema and altered gut formation and axial malformations. In *X. laevis* embryos, GLP and GBHs induced cephalic abnormalities, abnormal neural crest development, and anterior-posterior axis shortening, resulting in cranial cartilage deformities at the tadpole stages. Notably, the highest teratogenic indices indicated that Roundup and Enviro Glyphosate caused the most severe harm.^115^

GBH exposure similarly affected chicken embryos, leading to the gradual loss of rhombomere domains, decreased optic vesicles, and the development of microcephaly, which was linked to increased endogenous retinoic acid activity. These effects underscore the direct impact of GBHs on the early morphogenesis of the vertebrate nervous system.^116^ In contrast, a 52 week study in an avian model revealed that cumulative GBH exposure affected the overall composition of gut microbiota, suppressed the development of beneficial microflora, reduced hepatic catalase activity, and lowered male testosterone levels. However, reproductive physiology, including maturation, testis size, and egg production, remained intact.^117^ Regarding teratogenicity, perinatal oral exposure to GLP led to excessive lipoperoxidation and an overload of antioxidant enzyme systems in maternal and fetal serum and livers at 21 days of gestation.^118^ Transgenerational and multigenerational toxicity of orally administered GBHs was also reported. A study involving rat dams (F0) and two offspring generations (F1 and F2) revealed more pronounced effects in the F2 generation. While there were no changes in body weight or the onset of vaginal opening in the F1 offspring, the F2 offspring showed delayed growth, lower fetal weight and length, higher placental weight and placental index, and congenital morphological anomalies, despite a lower number of implantation sites.^119^

Furthermore, cesarean sections were performed on rat dams exposed to oral administration of Roundup from day 6 to 15 of pregnancy, revealing various outcomes, including corpora lutea, implantation sites, resorptions, and living and dead fetuses. Fetal examination confirmed external and skeletal malformations, while analysis of the dams showed numerous internal alterations and a high (50%) mortality rate among dams treated with 1000 mg/kg Roundup.^120^ Finally, the oral treatment of rat dams with Roundup during pregnancy (21–23 days) and lactation (21 days) adversely affected male offspring. That included a reduction in sperm production and quality during adulthood, a dose-dependent decrease in serum testosterone levels at puberty, and spermatid degeneration during both periods. Female offspring only exhibited a delay in vaginal canal opening.^83^

#### 3.1.4 Clinical Studies on GLP Impact on the Endocrine and Reproductive Systems

The influence of herbicides, pesticides, insecticides, fungicides, and fumigants on congenital disabilities among applicators’ children was also investigated (Figure 1). A population study involving 695 families and 1532 children conducted between 1997 and 1998 in the Red River Valley, Minnesota, revealed that the congenital disability rate was 31.3 per 1000 births in the first year of life and 47.0 per 1000 births within the first 3 years or later. A higher number of these defects were associated with conceptions in the spring. Notably, adverse neurologic and developmental neurobehavioral disorders (NBDs) were more prevalent among children of users of the phosphine fumigant and GBH.^121^ These findings align with in vivo data indicating a harmful link between sustained exposure of dams to the GBH MGB axis and the impairment of hippocampal neuroplasticity, learning, and memory, as well as the development of anxiety, autism-like behavior, and depression-like behavior in the offspring later in life.^25,122,123^ In neonate rats, gestational exposure to pure GLP led to dose-dependent NBDs in reflex development, motor activity, and cognitive functions, indicated by inhibiting the Wnt5a-CaMKII noncanonical signaling pathway.^124^ Finally, a miRNA microarray-based investigation of the association between GLP and NDDs in postnatal rats revealed upregulation of 55 genes and downregulation of 19 genes involved in the etiology of NDDs in the prefrontal cortex, particularly participating in neurogenesis, neuron differentiation, and brain development.^74^

Contradictory outcomes were reported by a meta-analysis investigating the association between human exposure to GMO GBH-treated corps in South America and reproductive system diseases, including congenital disabilities, abortions, preterm deliveries, childhood diseases, or altered sex ratios, as well as congenital malformations and disabilities. Except for attention-deficit hyperactivity disorder among children of GLP appliers, no significant associations were observed, which excludes the direct risk of human embryo- or teratogenicity of GLP or GBH.^125^

In contrast, the Ontario Farm Family Health Study (OFFHS), published in 1997 by the Canadian Census of Agriculture, provided evidence of the putative impacts of pesticides or herbicides, including GLP, on the human reproductive system. In this retrospective study, pesticide-exposed farm couples were surveyed about their farm activities, reproductive experiences, and occupational health risks. Particular attention was paid to the relationship between male health, within 3 months before conception through the month of conception, and miscarriage, preterm delivery, small-for-gestational-age births, and altered sex ratio. Identification of 3984 eligible pregnancies among 1898 couples (64% response) ruled out the significant association between male exposure to classified pesticides (including GLP) and the probability of small-for-gestational-age births or altered sex ratio. However, the combined use of various chemicals (GLP, atrazine, organophosphates, 4-[2,4-dichlorophenoxy] butyric acid, and insecticides) increased the risk of reproductivity complications and a continuation of the study focusing on miscarriage was strongly suggested.^126^

In the retrospective cohort evaluation, surveyed during 1991–1992, the OFFHS examined the influence of exposure to any of 13 pesticides on TTP. The 2012 planned pregnancies were analyzed in terms of the conditional fecundability ratio. In men’s exposure only to pesticide-related activity, three pesticides were associated with a 17–30% increase in fecundability. In contrast, six pesticides, including GLP, were associated with decreased fecundability in the women-only pesticide exposure case.^127^ According to another OFFHS study in 2001, targeting 2110 women who provided 3936 pregnancies, including 395 spontaneous abortions, preconception pesticide exposure to GBH (3 months before and up to a month of conception) was linked with a moderate risk of early abortion (<20 weeks) and an increased risk of late abortion.^128^

Direct association between exposure to GLP and TTP (measured in months) was assessed in 2592 fertile Colombian women from five regions exposed to different uses of GLP, applied by aerial spraying for illicit crop eradication. Retrospective interviews with the women regarding their reproductive health, life, and work, revealed no significant GLP effect on the TTP measured as fecundability odds ratios.^129^

In another birth-cohort study conducted in Central Indiana on 71 Caucasian women with singleton pregnancies, maternal GLP exposure was tested in terms of its pathological influence on exposure risk, frequency, and pathways as well as increased fetal exposure risk, fetal growth indicators, and pregnancy length. Liquid chromatography coupled with mass spectrometry (LC-MS) determination of urine and residential drinking water obtained from the subjected women showed GLP levels above the limit of detection of 0.1 ng/mL (the linear dynamic concentration range of 0.5–7.2 ng/mL) in 93% of the women, with a mean urinary GLP level of 3.4 ng/mL. In drinking water samples, GLP was undetectable. Although there were no correlations with fetal growth indicators, including birth weight and head circumference, an elevated GLP urine concentration was significantly correlated with shortened gestational length. However, despite geographical limitations and lack of racial and/or ethnic diversity, the study reported direct proof of the perinatal GLP exposure-associated threat on shortened pregnancy.^130^

Moreover, the impact of an abused GLP or GBH on human congenital disabilities was assessed. In the late 1990s, the relation of the selected congenital malformations occurrence upon occupational paternal exposure to pesticides was assessed in a case-referent study conducted in 8 hospitals of Comunidad Valenciana, Spain, with 261 matched pairs. No statistically significant associations between the father’s exposure to GBHs (including glufosinate) and the congenital disabilities in the first trimester of pregnancy were shown.^131^

In contrast, a cross-sectional study conducted in Red River Valley, Minnesota, U.S.A., during 1997–1998, among 1532 children of 695 pesticide applicator families, revealed unsettled data about the harmful impact of pesticides on the congenital disability rate. In the first year of life, this rate counted 31.3 births per 1000, with 83% of the total congenital disabilities reported by medical records. In the first three years of life or later, the rate increased to 47 per 1000. Neurologic and developmental NBDs refer to children of the applicators exposed to fumigant phosphine. NBDs were observed in children of the GLP group of the analyzed workmen.^121^

The association between maternal residential proximity (1000 m) and gestational exposure (month of conception) to 59 different agricultural pesticides and birth malformations was examined in infants with neural tube defects (NTDs), anencephaly, and spina bifida. In this two-control study, conducted in California in 1987–1991, the odds ratios were computed using conventional single- and multiple-pesticide models and hierarchical multiple-pesticide logistic regression in infants with NTDs. There was no association between GLP use and the NTD group in multiple-pesticide models. In contrast, an odds ratio was significant for the proximity to GLP and the occurrence of NTDs in the single-pesticide model. Elevated risks of NTDs, anencephaly, and spina bifida subtypes were also linked with carbamates, benzimidazole, and OPs.^132^

Infant cases of anencephaly (73), spina bifida (123), cleft lip with or without cleft palate (277), or cleft palate only (117) were subjects of another interview-based study that surveyed pregnant mothers who were residentially exposed to agricultural pesticide applications in San Joaquin Valley, California, in the years 1997–2007. As many as 35% of the interviewed mothers were threatened with the proxy activities of 52 chemical groups and 257 agricultural chemicals. However, there were no significant associations between maternal exposure during early pregnancy to GLP or GBHs crop spraying and these infant malformations.^133^

Researchers in a study conducted in the same geographical region examined 156 cases of infants and/or fetuses for pesticide-associated gastroschisis. A survey of 30 women exposed to GLP during pregnancy, among 22 pesticide groups and 36 specific pesticides, found no conclusive cause-and-effect link between agricultural exposure to GLP and gastroschisis.^134^ Gestational exposure to GLP/GBHs was not associated with a persistent cough, bronchitis, asthma, allergies, or hay fever in newborns, as observed in the analysis of 5853 pregnancies in the OFFHS study.^135,136^ Eventually, in the Agricultural Health Study (AHS) conducted in Iowa and North Carolina from 1993 to 1997, researchers examined 2246 women pesticide applicators and their infants to investigate the association between maternal exposure to pesticide use and low birth weight. Only 3% of the infants had low birth weight (less than 2500 g), and no significant birth weight loss was attributed to early pregnancy exposure to GLP or GBHs.^137^

### 3.2 GLP Impact on the Cardiovascular System

Globally, ∼18 million people die yearly from cardiovascular diseases (CVDs). CVDs are a group of heart or blood vessel disorders. Major causal factors of the CVDs relate to inappropriate diet, GM malfunctions, stress,^138^ epigenetics,^139^ congenital heart defect,^140^ environmental pollution,^141^ substance abuse,^142^ warfare agents intoxication,^143−146^ and occupational agrochemical exposure.^147−150^

#### 3.2.1 Preclinical Studies

**Figure 2:**
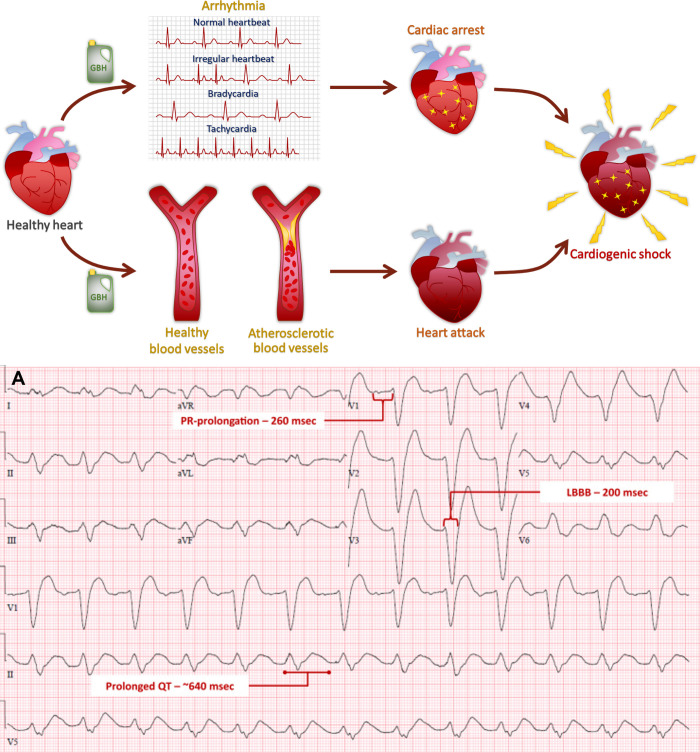
The GBH impact on the cardiovascular system. (A) Irregular electrocardiogram (ECG) on admission to the hospitalization of a 30-year-old woman who swallowed Roundup. The ECG, acquired approximately 4 h after syncope, shows sinus rhythm at 75 beats per minute with first-degree atrioventricular (AV) block (PR 260 ms), LBBB (QRS 200 ms), and significantly prolonged QT (670 ms). The patient recovered after 2 days of hospitalization. LBBB: left bundle branch block. Adapted with permission from ref (161). Copyright Elsevier 2019.

Cardiotoxicity of GLP, GLP-SH, and GBHs was investigated in vivo and in humans (Figure 2). In vivo studies confirmed the aggravating contribution of these agents to CVDs associated with developmental heart toxicity, GM dysregulation, arrhythmias, atherogenicity, and ventricular or aortic malformations. Remarkably, exposure of zebrafish embryos to a GLP solution caused structural abnormalities in the atrium, ventricle, and body vasculature, irregular heart looping, situs inversion, and a decrease in the heartbeat rate. Moreover, in situ hybridization and Mef2/mef2ca immunohistochemistry, performed during early cardiac patterning stages, confirmed the deviation of cardiomyocytic development.^151^ In contrast, a comparative study on the cardiotoxicity of GLP and Roundup, conducted on guinea pig hearts and human cardiomyocytes, confirmed the proarrhythmogenic properties of Roundup. At a relatively high concentration of 100 μM, Roundup significantly affected heart rate and reduced ventricular contractility and cardiomyocytic viability. In molecular terms, Roundup’s depressive impact on contractility was caused by concentration-dependent blocking of the CaV1.2 cardiac calcium channel. No such impacts of 100 μM GLP were observed, excluding the cardiotoxic properties of GBH’s adjuvants.^152^ Similarly, studies on rat and rabbit adults and offspring excluded developmental cardiotoxicity and cardiovascular malformations related to the GLP treatment applied during pregnancy.^153^ However, studies in vivo and in humans affirmed putative GLP- or GBH-related risk of atherosclerosis and tachycardia. In rats, 75-day-long oral and inhalation exposure to GLP in three concentrations resulted in a fatty streak, as demonstrated by histopathological examination. GLP exhibited a clear atherogenic potential. However, there was no dose- and exposure route-dependent alteration of the right and left ventricle thicknesses or in the collagen density.^154^ Oral administration of GLP and Roundup significantly increased the urinary level of homocysteine, a risk factor for CVD, related to a deviated *Prevotella* sp. abundance in the gut.^53^ Moreover, electrophysiological analysis of rats and rabbits treated with GLP and GBH showed electrical abnormalities, presumably resulting from a Roundup superfusion-induced reduction of intracellular calcium uptake. Beyond this excitability alteration, Roundup increased the incidence of arrhythmias in a dose-dependent manner. Nonetheless, a control group treated with GLP alone showed none of the above symptoms. This result suggests that, most likely, GBH surfactants and adjuvants, but not GLP itself, may cause life-threatening QT (ventricular repolarization) prolongation, atrioventricular conduction blocking, and arrhythmias.^155^

#### 3.2.2 Clinical Studies

Clinical studies on GLP/GBH intoxication primarily refer to evaluation of occupational risk in farmlands and herbicide factories. Alongside the plasmatic and urine GLP level determination, the QT prolongation was suggested to be monitored as the most common symptom of GBH intoxication to ascertain the risk of cardiovascular disease among farmers and GBH-factories workers.^156^ Prolonged PR intervals (called first-degree atrioventricular block) also belong to these symptoms. Prolongation of the PR interval, denoting the time from the beginning of atrial depolarization to the onset of ventricular depolarization,^157^ was analyzed in patients exposed to GLP herbicide formulations, including GLP ammonium salt herbicides and glyphosate isopropylamine (GLP-IPA) salt herbicides. As reported, of the two groups, GLP-IPA poisoning caused more fatality because of a higher incidence of QT prolongation and a higher tendency for PR prolongation.^158^ The QT interval was evaluated via a retrospective cohort study of 153 patients with acute GLP-SH ingestion as an early predictor factor for predicting mortality from GLP-SH intoxication. The 19 fatal cases were reported. A comparison of the electrocardiograms revealed that the nonsurvivors’ QT intervals were significantly longer than survivors, followed by intraventricular conduction and first-degree atrioventricular block.^159^ Likewise, a retrospective analysis of 232 GLP-SH-poisoned patients, including 29 deaths, showed significantly increased levels of lactate in nonsurvivors when compared to survivors. Additionally, this increase was markedly associated with 30 days of mortality, altered levels of potassium, and a prolonged QT interval. These findings suggest the usefulness of acidosis levels and QT interval measurements in the early prognosis of GBH-linked CVDs.^160^ Similarly, GLP cardiotoxicity mirrors acute sodium channel blocker overdose, leading to cardiogenic syncope, symptomized by diffuse electrophysiological depolarization and repolarization conduction abnormalities, including prolonged QTc, intraventricular block, and AV conduction delay (Figure 2). An electrocardiogram examination of a 30-year-old woman exposed to high-concentration GLP revealed that the exposure caused a syncopal episode in the left bundle branch block that evolved into a type I Brugada pattern and life-threatening arrhythmia^161^ (Figure 2A).

GBH hemotoxicity was encountered in the clinical cases of acute GBH poisoning. In 2013, Roundup, Pistol EV, Glyper, Grivolax, Verdis, or their mixtures, were used in 13 cases of suicide attempts, symptomized with oropharyngeal ulceration, nausea and vomiting, acidosis, respiratory issues, cardiac arrhythmia, hyperkalemia, impaired renal function, hepatic toxicity, and altered unconsciousness. In fatal cases, characterized by the 4146 mg/L GLP blood concentration (range of 690–7480 mg/L), cardiogenic shock, cardiorespiratory arrest, hemodynamic disturbance, and intravascular disseminated coagulation were dominated.^162^ Moreover, accidental and deliberate oral ingestions of GLP-trimesium (Touchdown) caused the deaths of a 6-year-old boy and a 34-year-old woman, respectively. The post-mortem examination revealed cardiopulmonary alterations, including edema and erosion of the mucus membranes of the airways and gastrointestinal tract, pulmonary and cerebral edemas, and deformation of the right atrium and ventricle of the heart.^163^ Moreover, refractory respiratory failure and cardiogenic shock were fatal in the suicidal case of a 57-year-old woman who died from swallowing a GLP-SH.^164^ Furthermore, a 65-year-old woman suffered from hyperkalemia, hypoxemia, and hypotension after the accidental ingestion of GLP-surfactant. The intoxication symptoms included increased creatinine levels, acute kidney injury, hemoconcentration, bicarbonate and lactate acidosis, and pneumonitis. The patient was detoxified using continuous hemofiltration and direct hemoperfusion.^165^ Moreover, persistent ventricular tachycardia and metabolic acidosis developed in a 47-year-old GLP-SH-poisoned man who recovered after extracorporeal membrane oxygenation was applied within 4 h of the cardiopulmonary collapse.^166^ Similarly, a 52-year-old man, intoxicated with GLP-POEA, experienced circulatory shock and refractory hypotension. Despite the nonresponsiveness to vasopressors, the patient recovered after a 5-h-long intravenous (I.V.) fat emulsion treatment,^167^ one of the most efficient therapies verified in a clinical survey, which included 64 patients.^168^

### 3.3 Lung (Respiratory) Diseases

#### 3.3.1 Preclinical Studies on Lung (Respiratory) Diseases

Lung diseases refer to pathologies of airways, air sacs, and vascular and neuromuscular elements of respiration, leading to airway obstruction, lung compliance, and the blockage of gas exchange. Major causative factors of respiratory diseases include autoimmune risks, allergens, infections, sepsis, cold, burns, smoking, air pollution, heavy metals, coal dust, asbestos, combat gases, and persistent exposition to agrochemicals.^169−182^

**Figure 3:**
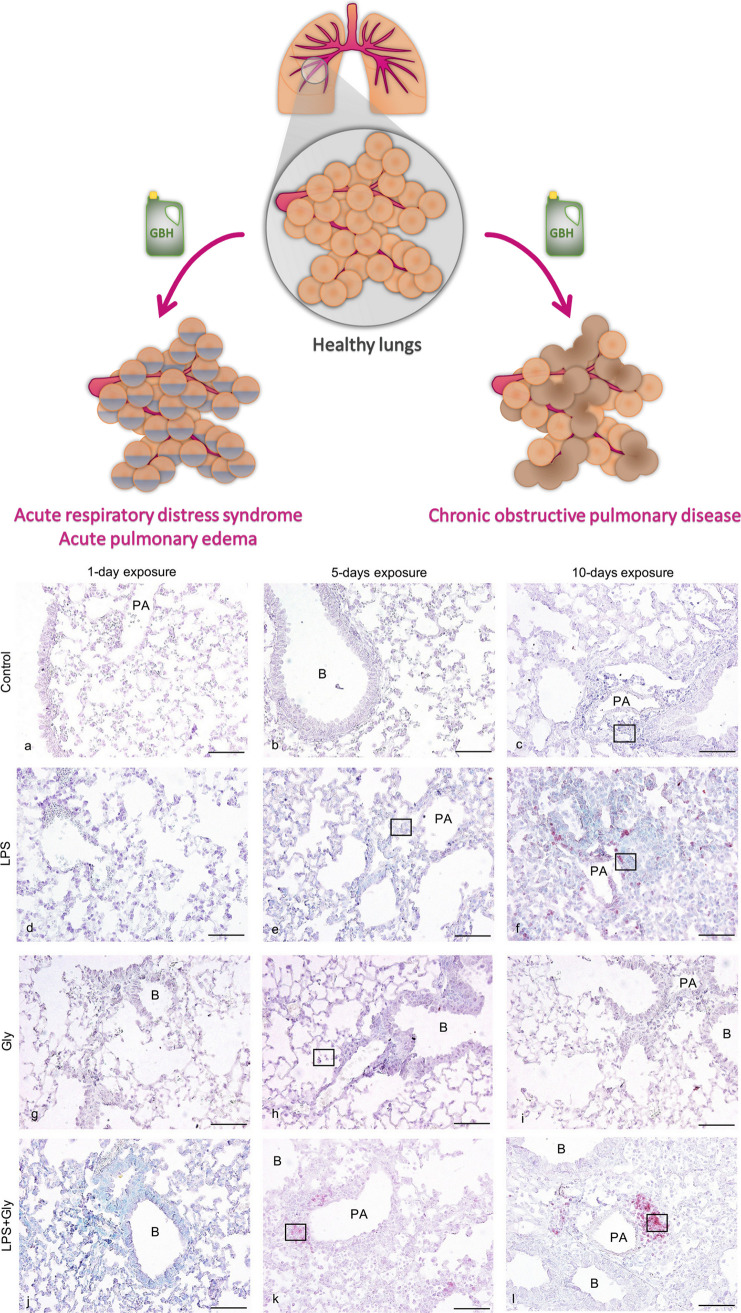
The GBH impact on the pulmonary system. (A–L) Immunohistochemical staining of T lymphocytes on mice lung tissue using CD-3 antibody, a pan-T lymphocyte marker. Expression of T lymphocytes in lung sections exposed for 1, 5, and 10 days to (A–C) control, (D–F) LPS (lipopolysaccharide), (G–I) GLP, and (J–L) LPS and GLP combination. The most intense T lymphocyte expression around lung perivascular regions (rectangles) was detected after (K) 5- and (L) 10-day exposure to the LPS and GLP combination. (H, I) No impact of pure GLP. Magnification ×400, scale bar 50 μm. B – bronchus; PA – pulmonary artery. Adapted with permission from ref (188). Copyright Elsevier 2021.

Environmental or occupational pesticide exposure’s most common pulmonary symptoms include cough, wheezing, dyspnea, breathlessness, chest tightness, chills, fevers, and sweats. Regarding occupational disorders, asthma, chronic bronchitis, chronic obstructive pulmonary disease (COPD), and pneumonia are most frequent among agricultural workers.^180^ Herbicide exposition-related lung disease case studies mentioned asthma, COPD, acute fibrinous and organizing pneumonia, pulmonary fibrosis, and lung cancer, as reviewed elsewhere^181,182^ (Figure 3). Mechanisms of agrochemical-caused respiratory pathophysiology associated with oxidative stress, inhibition of the parasympathetic system followed by airway hyperactivity, immunological alterations, including macrophage infiltration and eosinophil abscesses, and allergic response.^180−182^ The risk of agricultural airborne exposure to GLP involves inhaling the GLP containing eroded sediments and dust. Granulometric extraction of loess soil uncovered that GLP and AMPA were highly concentrated in soil particles of micrometer size, which positively correlated with clay, organic matter, and silt. The median half-life of GLP in the soil is between 2 and 197 days. Since the GLP decay in the soil is slow because of low soil moisture content, the health risk of off-site GLP inhalation increases significantly, which enhances the GLP airborne toxicity.^183,184^

Increased airborne concentration and slow dissemination in the soil of GLP may affect ground cover vegetation, e.g., impair immunity and the population of insects. GLP insectotoxicity was demonstrated for two evolutionary-distant species: *Galleria mellonella* (butterfly) and *Anopheles gambiae* (mosquito). Mechanisms underlying this activity were linked with GLP-inhibited melanization, i.e., the production of melanin, a black pigment involved in UV protection, thermoregulation, reactive species scavenging, and antimicrobial immunity. The GLP exposure indirectly attenuated insect immunity against *Cryptococcus neoformans*, a major fungal pathogen causing meningoencephalitis, and the *Plasmodium falciparum* parasite. Besides, GLP decreased the size of melanized nodules in the butterfly hemolymph and perturbed the midgut microbiome of the mosquito.^185^ The mechanistic association between life-threatening infection with *C. neoformans* and GLP-inhibited melanization was confirmed in mice.^186^ GLP-induced pulmonary pathology and inflammation were explored by measuring murine models’ cellular and humoral responses and lung functionality that inhaled GLP-enriched air samples collected on herbicide-sprayed farms. The GLP-rich air and GLP alone inhalation increased the level of eosinophil and neutrophils, mast cell degranulation, and TSLP and interleukin (IL) IL-33, IL-13, and IL-5 production. Both samples induced pulmonary (IL-13)-dependent inflammation and promoted Th2-type cytokine, providing evidence of a risk of GLP-induced occupational respiratory disorders.^187^ Moreover, proinflammatory outcomes of the GLP treatment were evaluated in the presence of endotoxin (lipopolysaccharide, LPS), a potent inflammatory agent. Levels of neutrophils, myeloperoxidase, tumor necrosis factor-α (TNF-α), IL-6, ICAM-1, and TLR-2 expression in mice exposed to the LPS-GLP comintation were higher than in mice exposed to either of these agents alone^188^ (Figure 3A–3L). Respiratory toxicities of GLP, POEA, a GLP-POEA mixture, and Roundup were compared in rats by using intratracheal administration. The POEA-containing preparations elicited a more rapid and prolonged respiration effect than the preparation with GLP alone. Noteworthy, within 1 h of treatment, all preparations appeared fatal. However, the mortality of the POEA preparations was higher than that of the GLP group.

Additionally, oral administration of POEA-containing preparations resulted in diarrhea and blood-stained weeping from noses, whereas animals of the GLP group expressed only diarrhea. Only 24-h treatment with POEA ended with death. Oral or intratracheal exposure to POEA and GLP caused lung hemorrhages and lung epithelial cell damage.^189^ Finally, respiratory disturbances caused by GLP-SH exposure were verified in humans. As reported in a clinical case report, oral intoxication with GLP-SH caused a blood pressure drop, metabolic and respiratory acidosis, respiratory distress, hypoxia, and altered consciousness. Further hospitalization uncovered sinus tachycardia, cardiomegaly free hilar congestion, and eventually acute pulmonary edema and respiratory failure.^190^

#### 3.3.2 Clinical Cases

Pulmonary and respiratory disorders are major symptoms of human death triggered by GBH poisoning. For example, in a suicidal case of GLP-SH ingestion, typical chemical pneumonitis and respiratory failure were associated with acute pancreatitis, which developed on the first day and lasted for 10 days.^191^ Moreover, in a clinical investigation conducted during 1992–1996, 36 out of 53 patients exhibited aspiration pneumonitis-associated laryngeal and mucosal injuries, which were considered potentially life-threatening.^192^ Finally, a case of GLP-SH-involved suicidal attempt of a 52-year-old woman reported aspiration pneumonitis and intestinal ileus. After the recovery, the woman suffered from a sudden upper-airway obstruction originating with fibrinous laryngotracheobronchitis.^193^

### 3.4 Liver Diseases

**Figure 4:**
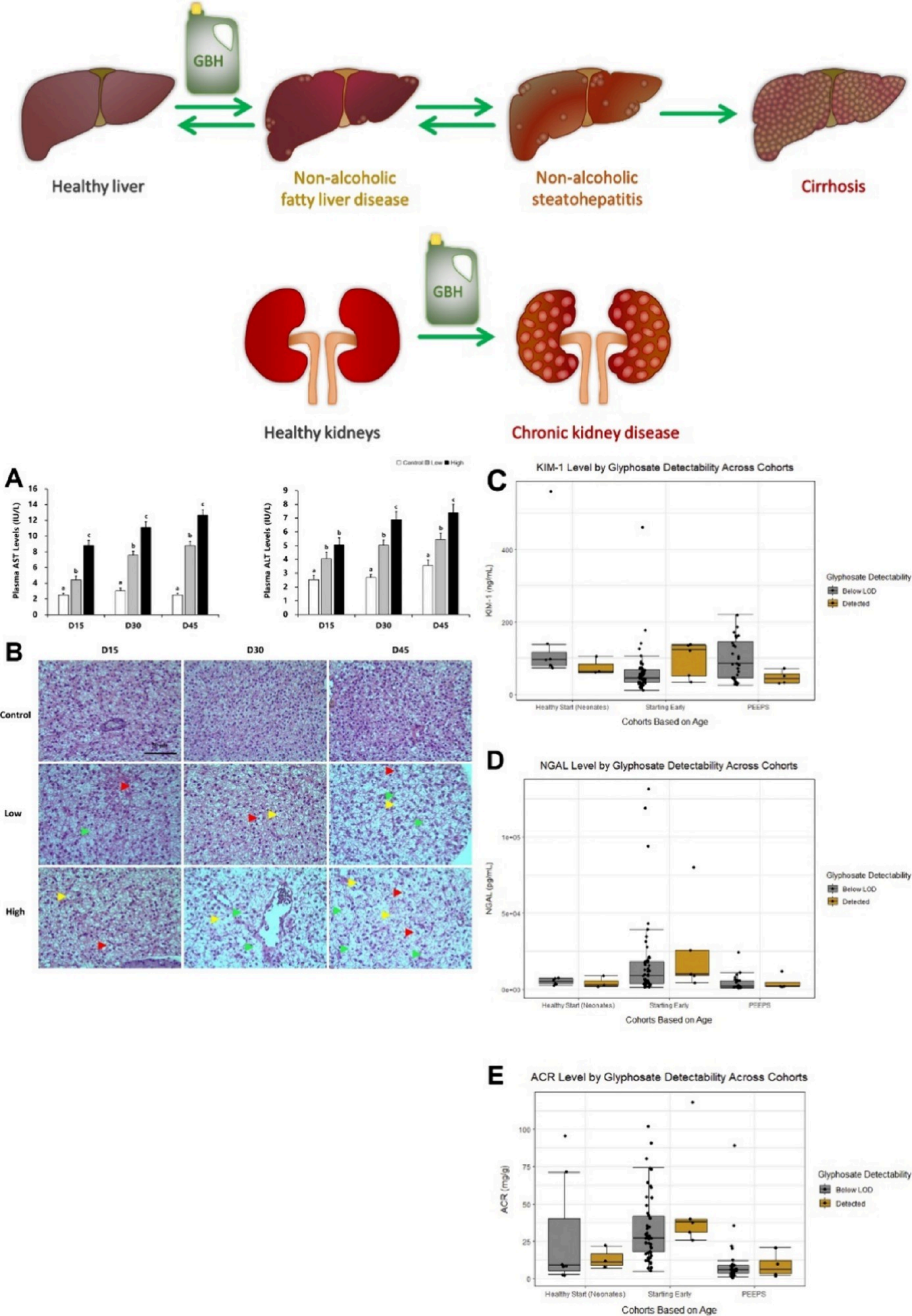
The GBH impact on the liver and the kidneys. (A, B) Assessment of GBH hepatotoxicity in common carp treated with GLP (0, 5, and 50 mg/L) for 45 days. (A) Activities of alanine transaminase (ALT) and aspartate transaminase (AST) in plasma samples collected after 15, 30, and 45 days of exposure. (B) Hematoxylin and eosin staining of liver sections to assess histopathological changes. Red, yellow, and green arrows indicate respective hepatocyte swelling, cytoplasmic vacuolation, and increased fatty changes. Adapted with permission from ref (196). Copyright Elsevier 2021. (C–E) Assessment of GLP nephrotoxicity in human urine samples. The dot plots illustrate the urinary levels of (C) kidney injury molecule (KIM-1), a renal tubular injury biomarker, (D) neutrophil gelatinase-associated lipocalin (NGAL), an acute kidney injury biomarker, and (E) albumin-to-creatine ratio (ACR), a biomarker of albuminuria, in participants enrolled in three different studies: Healthy Start, Starting Early, and Preventing Environmental Exposures in Pregnancy Study (PEEPS). Despite GLP detectability in urine (limit of detection of 0.1 ng/mL) of 11.1% of children at various ages, the multivariable regression models excluded the significant associations of these GLP exposures with any kidney injury biomarkers. Adapted with permission from ref (227). Copyright Elsevier 2020.

Liver (hepatic) diseases account for ∼2 million deaths per year globally.^194,195^ These pathologies’ primary mechanisms refer to hepatic inflammation, oxidative DNA damage resulting from infection, and obesity as well as alcohol, pharmaceutical, and drug abuse. Many controversies have arisen about whether GBP toxicity relates to formulants (surfactants, e.g., POEA and heavy metals) or GLP itself^196,197^ (Figure 4A and 4B). In vitro studies on the destructive impact of GBH in hepatoblastoma (HepG2), adenocarcinoma (A594), and neuroblastoma (SH-SY5Y) cell lines revealed that the ethoxylated formulants and their mixtures with GLP-IPA salt significantly inhibited proliferation of these cells, whereas GLP, the ActI, alone was not cytotoxic at all.^198^

Moreover, contradictory in vivo results were demonstrated. Pure GLP was hepatoxic to wall lizards (*Podarcis siculus*), important predators of herbivorous insects. Oral administration of low doses of GLP to a lizard caused fibrotic formation and a loss of liver functions. These malfunctions were associated with oxidative stress, manifested by the dysregulation of Cu/Zn superoxide dismutase, GSH-Px, metallothionein, and tumor suppressor protein 53, and upregulation of ERα and vitellogenin, thus showing the xenoestrogenic activity of GLP.^199^ Studies on rats confirmed GLP and GBH hepatotoxicity. 28-day feeding rats with GLP caused weight loss, triggered primary DNA damage in the liver cells and leukocytes, lowered thiobarbituric reactive substances in the liver and plasma, and dysregulated AChE and GSH-Px activity in the liver and plasma.^200^ Two-year chronic exposure to ultralow (0.1 ppb) Roundup, administrated via drinking water, occurred as hepatoxic and nephrotoxic. Transcriptome microarray analysis confirmed the GBH-related disruption of spliceosome and chromatin, lipotoxicity, phospholipidosis, and abnormal enlargement and necrosis of the liver and kidney cells associated with anatomical symptoms, including fibrosis and ischemia.^201^ Similarly treated rats had symptoms of steatohepatitis. The proteome analysis confirmed the GBH-induced disturbance of organonitrogen metabolism and fatty acid beta-oxidation, hallmarked by peroxisomal proliferation, steatosis (fatty liver disease), and necrosis. The metabolome analysis confirmed lipotoxicity and oxidative stress related to the glutathione and ascorbate free radical scavenger system. Likewise, the progression of steatohepatitis was associated with the GBH-induced alteration of biomarker levels of the nonalcoholic fatty liver disease biomarkers.^202^ Finally, the hepatotoxicity of GLP and Roundup has been confirmed in metagenomics and metabolomics profiling-based studies of rats exposed to these herbicides. These exposures caused markedly increased levels of gastrointestinal, hepatic, and oxidative stress biomarkers associated with shikimate and 3-dehydroshikimate, reflective of the inhibition of EPSPS of the shikimate pathway. These outcomes suggest a severe herbicidal impact on rat gut microbiota, although it must be highlighted that Roundup’s toxicity was higher when compared to GLP.^203^ Particularly, regarding liver biochemistry, the multiomics approach confirmed a herbicidal-caused deviation of nicotinamide (vitamin B_3_) metabolism that naturally prevents hepatic steatosis by increasing the redox potential.^204^ These results were reinforced in a comparative 12-month study on hemo- and hepatotoxicity of GLP and Roundup in rabbits, designed for the real-life risk simulation (RLRS) approach. Toxicities of GLP and Roundup were evaluated versus a mixture of common endocrine disruptors and xenobiotics containing GLP, bisphenol, and triclosan as well as phthalates and paraben derivatives. As a result, GLP displayed only redox perturbations in blood homeostasis, whereas there were no effects of GLP on liver tissue. This relatively minor outcome was contradictory to the effects of Roundup and a mixture of endocrine disruptors that distorted blood redox equilibrium and caused oxidative stress manifested by decreases in the activities of SOD and glutathione reductase and increases in the total antioxidant capacity and activities of GSH and GSH-Px. Overall, these findings confirmed the RLRS approach applied to the hemo- and hepatoxicity of Roundup and common xenobiotics, whereby the harm of pure GLP was the lowest or negligible.^205^

Additionally, the subacute exposure of rats to Roundup caused adverse inflammatory effects. The Roundup treatment elevated levels of C-reactive protein, cytokines IL-1β, IL-6, TNF-α, and prostaglandin-endoperoxide synthase in the liver and adipose tissue. These results correlated with histological analysis showing the formation of vacuoles, fibroid tissue, and glycogen depletion, thus suggesting the progression of fatty liver disease, multiorgan inflammation, and liver scarring.^206^

Preclinical studies uncovered GLP- and GBH-induced hepatotoxicity, congestive hepatopathy, and liver fibrosis. These disorders were confirmed in patients with nonalcoholic steatohepatitis (NASH) and biopsy-determined nonalcoholic fatty liver disease (NAFLD), the most common chronic liver disease in developed countries nowadays^194,195^ (Figure 4). Particularly, high-performance liquid chromatography (HPLC) examination of urine profiles revealed a significant increase in GLP excretion in NASH patients compared with non-NASH patients. These results and a dose-dependent GLP exposure-fibrosis stage correlation suggest that NASH patients are more susceptible to fibrosis progression and the development of cirrhosis and hepatocellular carcinoma.^207^

Clinical cases of GLP-related hepato- and nephrotoxicity in humans mainly refer to commercial GBHs. As documented, multiple symptoms of GBH intoxication include respiratory failure, GI dysfunction, neurological disorders,^208^ and disruption of the liver and kidneys.^209^

### 3.5 Nephrotoxicity and Kidney (Renal) Disease

#### 3.5.1 Preclinical Studies

Kidney (renal) disease, also called nephropathy, results from inflammatory (nephritis) or noninflammatory (nephrosis) renal malfunction. Two significant types of kidney disease are distinguished, namely, chronic kidney disease (CKD), lasting longer than three months,^210^ and acute kidney injury, i.e., a sudden, severe impediment to renal function.^211^ In 2017, the global burden of CKD reached 1.2 million deaths, making it the twelfth leading cause of death (Figure 4). Major CKD causes include diabetes, hypertension, cardiovascular disease, xanthine oxidase deficiency, retention of analgesics or nephrotoxins, deposition of antibodies (glomerulonephritis), as well as lupus, sepsis, polycystic kidney disease, kidney stones, and infections of the urinary tract.^212,213^ In vivo toxicogenomic studies confirmed the nephrotoxic effects of the GLP and Roundup formulations. For example, hyaline cysts, mineralization pelvis, epithelial pelvis, kidney tubular fibrosis, and kidney tubular degeneration were observed in rats treated with these herbicides. These malformations were associated with genotoxic alterations and DNA damage in the kidney.^214^

#### 3.5.2 Clinical Studies

Epidemiological reports have hallmarked the pesticide- or herbicide-induced kidney diseases in farmers worldwide, including in Sri Lanka and India,^215−218^ and the U.S.A.^219,220^ A critical summary of global pesticide CKD epidemics, including GLP and GBH, was presented elsewhere.^221^ Noteworthy, the environmental use of GLP and GBH-related CDK risk had arisen since 1994 when GLP became a potential causal factor for CKD of unknown etiology in rice paddy farming areas in the dry zones of Sri Lanka (called Sri Lanka Agricultural Nephropathy). The putative GLP-induced CKD outbreak was associated with the GLP spraying, particularly with the consumption of hard water and exceptional metal chelating properties of GLP. As revealed by inductively coupled plasma mass spectrometry and ELISA analyses, samples of drinking water from serving wells and abandoned wells contained traces of GLP and metals, including Ca, Mg, Ba, Sr, Fe, Ti, and V.^215,216^ Besides, urine profiles of the farmer patients living in endemic areas revealed urinary Sb, As, Cd, Co, Pb, Mn, Ni, Ti, and V concentrations exceeding the reference ranges. Moreover, GLP and creatinine urinary levels were elevated compared to the nonendemic controls.^217^ Finally, LC-MS analyses of topsoil samples from agricultural fields, water samples from nearby shallow wells and lakes, and sediment samples from lakes confirmed the presence of GLP complexes with Fe and Al and strong retention of GLP in soil and groundwater.^218^ Nephrotoxicity of GLP-metal complexes is well documented by studies in vivo and in humans.^222−224^ In 2014, a clinical case report demonstrated airborne GLP-induced hepatorenal dysfunction in workers of GLP-producing factories.^225^ However, contradictory results were obtained by comparing GLP and Roundup renal toxicity in male rats. By measuring levels of kidney biomarkers, including serum urea and creatinine, plasma cystatin-C and neutrophil gelatinase-associated lipocalin (NGAL), and oxidative stress indices related to activities of several kidney membrane-bound enzymes, we showed that Roundup-exposed rats accumulated more xenobiotics (including GLP, the ActI) than the group exposed to GLP alone. This increased accumulation was associated with nephrotoxicity, hallmarked by deviated levels of the biomarkers, whereas GLP administrated alone displayed no effect on renal function.^226^ Eventually, GLP potential nephrotoxicity was evaluated in children by measuring urinary levels of kidney injury biomarkers including albuminuria, NGAL, and kidney injury molecule-1. Despite GLP detectability in the children’s urine, there was no evidence of GLP-induced renal injury^227^ (Figure 4C–4E).

Clinical cases of GBH intoxication involve severe GBH nephrotoxicity.^228^ In the case of an intentionally intoxicated 22-year-old man, the poisoning caused a cute hemolysis, acidosis, and compensatory respiratory alkalosis. Besides, the GLP-SH increased the permeability of the erythrocyte membrane by disturbing the lipid bilayer and consequent hypotonic hemolysis, which resulted in multiorgan collapse, including frequent ventricular tachycardia, acute renal failure, rhabdomyolysis, coagulation dysfunction, and urinalysis. The patient recovered after alkaline diuresis, emergency plasmapheresis, and blood component transfusion.^229^

### 3.6 Cancer

Considerable controversy regarding cancer risk associated with both the use and misuse of GBH has recently arisen among scientists, authorities, and society.^230,231^ Chronologically, in 2014, EFSA classified GLP as “unlikely to pose a carcinogenic hazard to humans”. A year later, the International Agency for Research on Cancer (IARC) stated that GLP is a “probable human carcinogen” (Group 2A),^232^ whereas, in 2016, the EPA concluded that it is “not likely to be carcinogenic to humans”.^233^ In 2017, the European Chemical Agency (EChA) denied a support link between GLP and animal cancer.^234^ However, the Joint Meeting of Pesticide Residues (JMPR) agreed on the possibility that GLP “is cancerogenic in mice at very high doses”.^235^ In 2018, the AHS cohort study declined any association of GLP with any solid tumors or lymphoid malignancies, including non-Hodgkin lymphoma (NHL) and its subtypes, with only some evidence of increased risk of acute myeloid leukemia.^236^ These discrepancies originate from various sources of epidemiological data, lack of established criteria for statistical analyses and meta-analyses, overconfidence in common cancer etiology in experimental animals and humans, as well as differences in toxicological impacts and human incidence exposures to pure GLP and GBH formulations.^231,236−238^ Despite clear GLP-induced cancer cases in rodents, including hemangiosarcomas, hemangiomas, kidney and liver adenomas, malignant lymphomas, NHLs, skin keratoacanthomas, adrenal cortical carcinomas, and skin basal cell tumors, summarized in 2020,^231^ some of these reports have already been questioned.^238^ Although the recent epidemiological evidence has recalled the carcinogenic potential of GLP in humans,^239^ the GLP-induced oxidative damage, chromosomal alterations in human lymphocytes, and stimulation of cell proliferation, i.e., the major causative factors of cancer, cannot be underestimated.^240−245^ These results are consistent with comparative toxicogenomics conducted on rats exposed to GLP and three Roundup formulations used in the EU (MON 52276), United Kingdom (Roundup ProBio, MON 76473), and the United States (Roundup PROMAX, MON 76207). Generally, these herbicides caused formulation- and organ-specific genotoxicity in the liver and kidney. Significant carcinogenesis-associated alterations were observed in the epigenome (differences in CpG methylation and levels of miR-10, miR-17, miR-22, and miR-30), DNA damage-associated TP53 protein activation, deviated circadian rhythm regulation, oxidative stress, and unfolded protein response. Importantly, Roundup formulations were far more genotoxic than GLP. However, GLP, in particular, caused apurinic/apyrimidinic DNA damage in the liver.^214^ In this context, worth mentioning is that the augmented risk of cutaneous melanoma associated with occupational exposure to the sun, GBH, and fungicides was alerted among human subjects in Italy and Brazil.^246^

## 4 Theranostics of Glyphosate Intoxication

GBH intoxication triggers two major pathological clinical effects. First, it affects the GM and impacts the MGB and HPA axes. This dysbiosis increases GI motility, resulting in fecal and urinary incontinence, miosis, diaphoresis, and diaphragmatic failure. Second, after entering the nervous system, GLP, an OP, phosphorylates the serine hydroxyl groups in AChE, thus inhibiting the hydrolysis of ACh, which causes dysregulation of cholinergic neurotransmission and overstimulation of muscarinic and nicotinic receptors in skeletal muscles.^254^

So far, no antidote has been developed for GLP-SH poisoning, and the therapy is mainly symptomatic and supportive.^255,256^ Pharmacological therapy supplies the use of competitive ACh antagonists or pseudoreversible inhibitors of cholinesterase. In the case of established OP exposure, therapy includes pretreatment.^254^ Carbamate pyridostigmine (the only FDA-approved substance for this pretreatment) is investigated most widely. However, the CNS remains unprotected because of its incapability to cross the blood–brain barrier (BBB).^27,257^ In typical therapy of OP poisoning, three FDA-approved therapeutics are used, vis., atropine, pralidoxime or obidoxime, and diazepam, among which atropine is the most common.^257^ Another group of therapeutics includes enzyme-based GLP inactivators. They enable GLP transformation and biodegradation in the bloodstream before the GLP crosses the BBB. The transformation rate constants span from 0.08 to 100 h^–1^ (butyrylcholinesterase) through 4–340 min^–1^ (paraoxonases) to 5–2100 s^–1^ (phosphotriesterase or organophosphorus hydrolase).^254^ Other therapeutics, including non-FDA-approved oximes, alkaloids, anti-NDMA agents, MgSO_4_ magnesium sulfate and NaHCO_3_, and antibody-enzyme-nanoparticle conjugates, have been reviewed elsewhere.^254^

HPLC-MS and ELISA have been employed in conventional medical diagnostics of GLP or GBH poisoning. They are mainly used for the toxicological determination and degradation of GLP in the blood and urine. Emergency treatment of acute GBH intoxication involves HDF- or DHP-assisted gastric lavage with a large amount of normal saline followed by active charcoal administration.^165^ Further detoxification includes charcoal hemoperfusion and pulse therapies of cyclophosphamide and methylprednisolone, followed by dexamethasone application. In cases of hypoxemia, glucocorticoid and cyclophosphamide pulse therapies are applied.^256^ Intensive care is required in severe GLP-SH intoxication, symptomized by dehydration, oliguria, paralytic ileus, hypovolemic shock, cardiogenic shock, pulmonary edema, hyperkalemia, and metabolic acidosis.^165,249^ In the case of hyperkalemia, the urine and blood concentrations of potassium, GLP, and AMPA can be decreased by an enema with polystyrenesulfonate^251^ or intragastric cathartic and charcoal administration.^252^

Pharmacological treatment is applied to injuries to the digestive system, including constipation and overall digestive peristalsis.^253^ However, colon resection and colostomy are the only solutions for mild colonic distention and peritonitis.^209^ In cases of hypotension, vasopressor-based therapy can be supported by hemodialysis,^164,250^ and I.V. lipid emulsion application.^167,168^ For multiorgan failure, including cardiopulmonary and acute kidney failure, extracorporeal membrane oxygenation^166^ or alkaline diuresis, emergency plasmapheresis, and blood component transfusion^229,258,259^ are recommended.

## 5 Conclusions and Future Prospective

Modern toxicology and epidemiology have been revolutionized by ultrasensitive analytical tools of precision (personalized) theranostics and wearable or smartphone-assisted artificial intelligence-excelled sensors or drug delivery systems.^260^ Regarding GLP poisoning and GLP above’s modes of actions, i.e., dysbiosis and the inhibition of AChE, these devices, electronically powered, decision-making, and user-friendly, shall enable self-handled or point-of-care professional-assisted evaluation of the harm followed with rapid and precise determining and removing the herbicide and coformulants. In technological terms, these devices will be constructed as biocompatible chemo- and (bacteria cell)-based electronic biohybrids and nanorobots, capable of measuring the GBH analyte or GBH pathology-associated biomarkers in skin, when implanted or wearable as a chip, in body, when swallowed, or in body fluids liquid, when used as a portable, smart device.^19,261−263^ Finally, it is expected that chemisorptive or adsorptive tissue-specific systems will be manufactured to detect and capture the GBH xenobiotics with molecular recognition-provided selectivity and sensitivity, which will be followed by harmless, on-site, and nature-mimicking microbial biodegradation of GLP.^264,265^

## List of Abbreviations

ACh: acetylcholine
AChE: acetylcholinesterase
AHS: Agricultural Health Study
ActI: active ingredient
AMPA: (aminomethyl)phosphonic acid
AR: androgen receptor
AV: atrioventricular (block)
BBB: blood–brain barrier
circRNA: circular ribonucleic acid
CKD: chronic kidney disease
COPD: chronic obstructive pulmonary disease
CVD: cardiovascular disease
DHP: direct hemoperfusion
DNA: deoxyribonucleic acid
ECETOC: European Centre for Ecotoxicology and Toxicology of Chemicals
EChA: European Chemical Agency
EDC: endocrine disrupting chemical
EDSP: Endocrine Disruptor Screening Program
EFSA: European Food Safety Authority
ELISA: enzyme-linked immunosorbent assay
EPA: Environmental Protection Agency
EPSPS: 5-enolpyruvynyl-shikimate-3-phosphate synthase
ER: estrogen receptor
ESD: endocrine-system disruptor
FAO: Food and Agriculture Organization
FSH: follicle-stimulating hormone
GBH: glyphosate-based herbicide
GE: genetically engineered (organism)
GI: gastrointestinal
GLP: glyphosate
GLP-IPA: glyphosate isopropylamine
GLP-SH: glyphosate surfactant
GM: gut microbiota (microbiome)
GMO: genetically modified organism
GnRH: gonadotropin-releasing hormone
GSH-Px: glutathione peroxidase
HDF: hemodiafiltration
HOXA: homeobox protein Hox-A10
HPA: hypothalamic-pituitary-adrenal (axis)
HPLC: high-performance liquid chromatography
HPO: hypothalamic-pituitary-ovarian (axis)
HPP: hypothalamic-pituitary-peripheral glands (axes)
HPT: hypothalamic-pituitary testes (axis)
HPTh: hypothalamic-pituitary thyroid (axis)
I.V.: intravenous (injection)
IL: interleukin
IARC: International Agency for Research on Cancer
JMPR: Joint Meeting of Pesticide Residues
KCs: key characteristics
LC: liquid chromatography
LC-MS: liquid chromatography coupled with mass spectrometry
LH: luteinizing hormone
LPS: lipopolysaccharide
MDA: malondialdehyde
MGB: microbiota–gut–brain
miRNA (miR): microribonucleic acid
mRNA: messenger ribonucleic acid
MS: mass spectrometry
NAFLD: nonalcoholic fatty liver disease
NASH: nonalcoholic steatohepatitis
NBD: neurobehavioral disorder
NDD: neurodevelopmental disorder
NGAL: neutrophil gelatinase-associated lipocalin
NHL: non-Hodgkin’s lymphoma
NTD: neural tube defect
OECD: Organization of Economic Co-operation and Development
OFFHS: The Ontario Farm Family Health Study
OP: organophosphate
POEA: polyoxyethyleneamine
PND: postnatal day
PR: first-degree atrioventricular block (interval)
ProgR: progesterone receptor
QT: time between the start of the Q-wave and the end of the T-wave in the electrocardiogram
RLRS: real-life risk simulation
ROS: reactive oxygen species
StAR: steroidogenic acute regulatory (protein)
TH: tyrosine hydrolase
TLR: toll-like receptor
TNF: tumor necrosis factor
TTP: time-to-pregnancy
WHO: World Health Organization
